# Development and Temporal Evaluation of a Tiered Clinical–Ultrasound Prediction Model and Simplified Integer Score for Time-Sensitive Pathology in Women with Acute Pelvic Pain: A Retrospective Cohort Study

**DOI:** 10.3390/diagnostics16183003

**Published:** 2026-09-16

**Authors:** Marijana Basta Nikolić, Dragan Vasin, Jelena Pilipović Grubor, Mirela Juković, Dijana Nićiforović, Dragan Nikolić, Sanja Stojanović

**Affiliations:** 1 Faculty of Medicine, University of Novi Sad, 21137 Novi Sad, Serbia; mirela.jukovic@mf.uns.ac.rs (M.J.); sanja.stojanovic@mf.uns.ac.rs (S.S.); 2 Center for Radiology, University Clinical Center of Vojvodina, 21000 Novi Sad, Serbia; jelkepilip@gmail.com; 3 Emergency Radiology Department, Center for Radiology, University Clinical Center of Serbia, 11000 Belgrade, Serbia; draganvasin@gmail.com; 4 Clinic for Vascular and Endovascular Surgery, University Clinical Center of Vojvodina, 21000 Novi Sad, Serbia

**Keywords:** acute pelvic pain, clinical prediction model, temporal evaluation, ultrasonography, risk score, bootstrap stability, calibration, decision curve analysis

## Abstract

**Background/Objectives**: Clinical prediction models may support staged triage in acute pelvic pain, but simplified integer scores can lose information and destabilise point assignments. We evaluated a tiered clinical–ultrasound prediction pathway and the stability of its simplified score within an ultrasound-documented acute-pelvic-pain cohort. **Methods**: This single-centre retrospective cohort included 4800 encounters in 4320 women. Encounters from 2013 to 2019 formed the development cohort (*n* = 3360), and patient-separated encounters from 2020 to 2022 formed the temporal-evaluation cohort (*n* = 1440). M1 contained clinical and laboratory predictors without imaging variables, M2 added ultrasound descriptors, and M3 added cross-sectional-imaging descriptors. Multiple imputation, ridge logistic regression, patient-level cluster bootstrap, calibration, decision-curve analysis, reader reproducibility and score-stability analyses were used. **Results**: Urgent pathology occurred in 20.0% of development and 18.0% of temporal encounters. Temporal AUC was 0.841 for M1 and 0.845 for M2 (difference 0.004; 95% CI 0.001–0.009). Because inclusion required documented ultrasound, this difference estimates incremental information from the selected ultrasound descriptors within an already ultrasound-documented pathway and does not estimate the pathway-level benefit of performing ultrasound. The locked integer rule classified 2.9% as low risk, with 99.6% sensitivity and 3.5% specificity. Categorisation accounted for 0.057 of the total 0.060 AUC loss, and 798 distinct score cards arose in 800 bootstrap resamples. **Conclusions**: Continuous M2 retained good temporal discrimination, but the small M1-M2 difference does not establish whether ultrasound improves pathway-level triage or can safely alter subsequent management. The integer score had low rule-out yield and highly variable point assignments; independent evaluation should prioritise local calibration, clinical utility and score-card stability before implementation.

## 1. Introduction

Acute pelvic pain is a common and diagnostically heterogeneous emergency presentation in women. Gynaecological, urinary, gastrointestinal and vascular disorders may produce overlapping symptoms and signs, and delays in recognising ectopic pregnancy, adnexal torsion, appendicitis, bowel ischaemia or obstruction, infected urinary obstruction and vascular emergencies can result in substantial morbidity. Imaging is therefore central to the diagnostic pathway. Ultrasonography is generally the first-line modality, while computed tomography (CT) and magnetic resonance imaging (MRI) are selected according to pregnancy status, suspected aetiology and the results of the initial assessment [1,2].

Clinical prediction models could support this staged pathway, but their predictors must be available at the point at which the model is intended to be used. A post-ultrasound model cannot justify the omission of the ultrasound examination that generated its inputs, and a CT/MRI descriptor cannot support a decision to avoid cross-sectional imaging. Prediction-model reporting and evaluation should therefore address not only discrimination, but also calibration, clinical utility, transport across time, missing data, measurement reliability and the stability of the final model representation [3,4]. Contemporary evaluation guidance places particular weight on calibration and decision-analytic assessment rather than discrimination alone [5,6,7].

Simplified integer scores are attractive because they can be applied without software. However, simplification creates additional statistical vulnerabilities. Categorisation of continuous predictors discards information [8], penalisation affects coefficient magnitude [9], sparse interaction terms can be unstable, and small coefficient changes may move a predictor across a prespecified rounding boundary. Conventional sample-size criteria are designed primarily to limit global overfitting and improve prediction accuracy; they do not guarantee reproducibility of every rounded point weight [10,11,12,13]. For a rule-out score, high sensitivity is also insufficient by itself: specificity, low-risk yield and the clinical interpretability of the selected threshold determine whether the rule can meaningfully change care.

We therefore evaluated a tiered clinical–laboratory and imaging triage modelling pipeline for time-sensitive pathology in women with acute pelvic pain. Our aims were to: (1) assess temporal discrimination, calibration, specificity, rule-out yield and decision-analytic utility across the attainable threshold range; (2) separate the discrimination loss caused by categorisation from that caused by integer rounding; (3) quantify prediction-level, classification-level and complete score-card stability, including the influence of a sparse interaction close to a rounding boundary; and (4) examine sensitivity to the missing-data strategy.

## 2. Materials and Methods

### 2.1. Study Design, Cohort and Ethics

We conducted a single-centre retrospective cohort study using electronic health record data from emergency and surgical/gynaecological triage services at the University Clinical Center of Vojvodina, Novi Sad, Serbia, from 1 January 2013 to 31 December 2022. Reporting followed TRIPOD + AI and STROBE [3,4]. A formal Statistical Analysis Plan was documented before data extraction and prespecified the primary outcome, 72 h window, predictor tiers, two interactions, clinical cut-offs, B = 0.40 scaling constant, and the rule selecting the highest integer threshold with development sensitivity ≥ 95%. The study was approved by the Ethics Committee of the Faculty of Medicine, University of Novi Sad (01-3920/2; 15 January 2025); informed consent was waived for retrospective use of routinely collected data.

**Figure 1:**
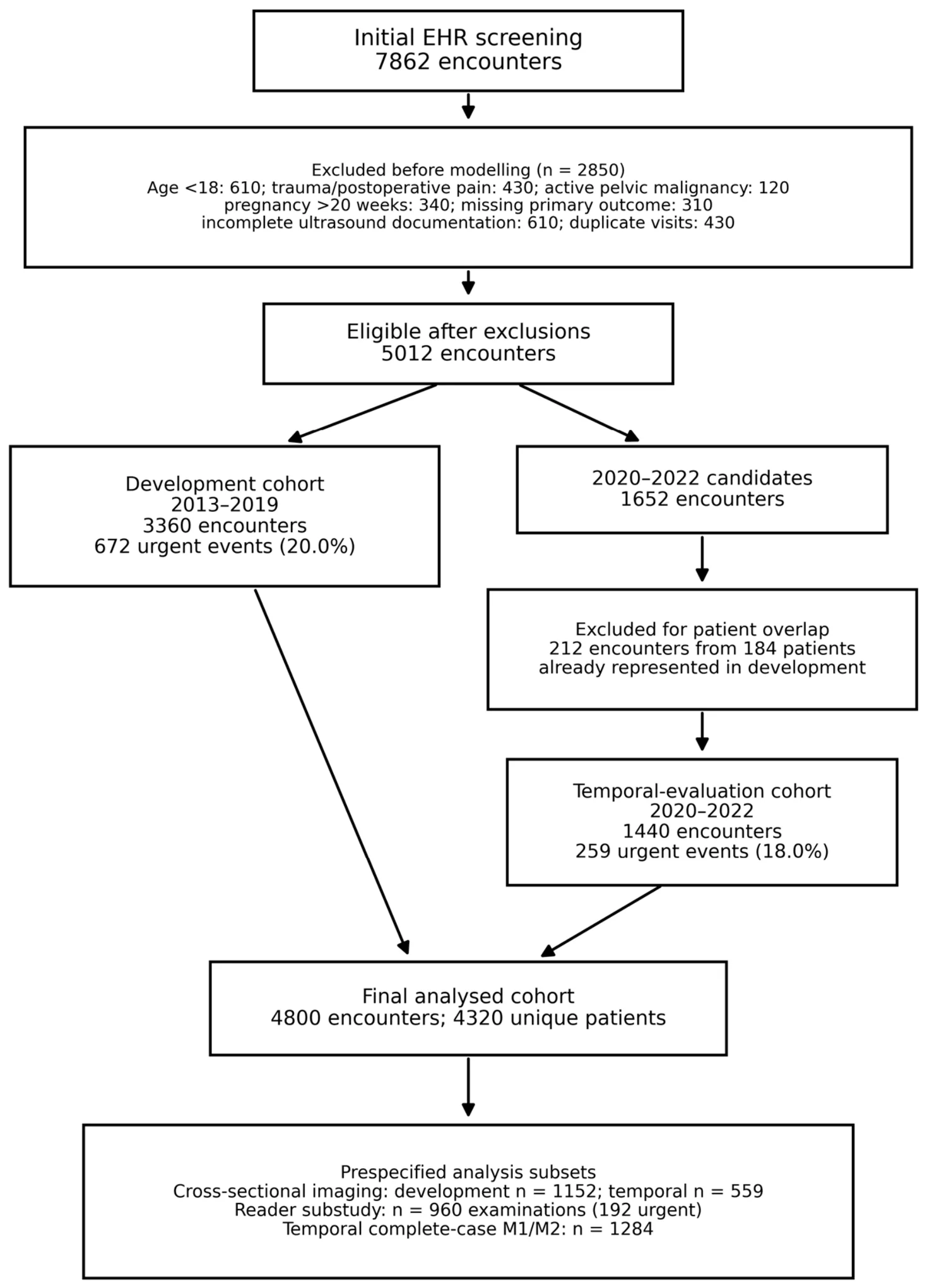
Cohort identification, strict temporal allocation and prespecified analysis subsets. Counts are encounters unless otherwise indicated. Temporal evaluation excluded 212 encounters from 184 patients already represented in the development cohort.

Initial screening identified 7862 encounters for acute non-traumatic lower abdominal or pelvic pain within 7 days of symptom onset. After the prespecified exclusions shown in Figure 1, 5012 encounters remained. Encounters from 2013 to 2019 formed the development cohort (*n* = 3360). To prevent patient-level temporal leakage, 212 encounters in 2020–2022 from 184 patients already represented in the development were excluded; the remaining 1440 encounters formed the temporal-evaluation cohort. The final dataset comprised 4800 encounters in 4320 unique women. The encounter was the prediction unit; recurrent eligible episodes were retained, while patient clusters were preserved in cross-validation and bootstrap procedures. Because complete ultrasound documentation was required for inclusion, the target population is an ultrasound-documented acute-pelvic-pain pathway rather than all unselected emergency presentations. Full cohort and extraction details are provided in Supplementary Methods S1.

### 2.2. Outcome and Predictor Information Stages

The primary outcome was time-sensitive pathology requiring therapeutic intervention or specialised inpatient management within 72 h of presentation. Two senior attending physicians (a gynaecologist and a general surgeon, each with >15 years of acute-care experience) independently adjudicated diagnoses from operative, histopathology, radiology, discharge and 30-day follow-up records; 42/931 (4.5%) urgent cases required resolution by a third senior emergency-radiology adjudicator. Because radiology reports contributed to both predictor extraction and adjudication, an alternative-outcome sensitivity analysis counted only operative- or histopathology-confirmed urgent outcomes as events (763/931, 82.0%), with imaging-only urgent cases retained in the denominator. Detailed outcome definitions and diagnosis mapping are reported in Supplementary Methods S2a and Table S8.

The benchmark model included age, continuous C-reactive protein (CRP), white blood cell count (WBC) and peritonism. M1 added positive beta-hCG, haematuria, temperature and shock index but no imaging variables; because retrospective timestamps did not establish that every laboratory result preceded ultrasound, M1 is interpreted as a non-imaging rather than a strictly pre-ultrasound model. M2 added five ultrasound descriptors (complex free fluid, absent/diminished ovarian flow, adnexal mass, tubal-wall thickening and hydronephrosis) plus a beta-hCG × complex-fluid interaction. M3 was an exploratory cross-sectional-imaging extension adding four CT/MRI descriptors and a haematuria × perinephric-stranding interaction; it was evaluated only in imaged encounters (development *n* = 1152; temporal *n* = 559). Operational definitions are in Supplementary Methods S2.

### 2.3. Reader Reproducibility and Missing Data

A temporal outcome-stratified substudy included 960 ultrasound examinations (192 urgent and 768 non-urgent) from 874 unique patients. Two radiologists independently reviewed archived static images and cine loops while blinded to outcome, routine reports, clinical records and each other. Agreement for the five M2 ultrasound descriptors was assessed using observed agreement and Cohen’s kappa [14]; each reader’s classifications were then substituted into the locked M2 coefficients without refitting. Because 79 patients contributed repeated examinations, reader-specific AUC 95% CIs were obtained with a patient-cluster bootstrap in which unique patients, rather than examinations, were resampled. The original examination-level DeLong intervals [15] were retained only as a descriptive sensitivity comparison in Supplementary Table S6a. Full sampling, blinding and 2 × 2 reader details are in Supplementary Methods S2b and Table S6a.

Missing M1/M2 predictors were handled primarily with multiple imputation by chained equations using 20 imputed datasets [16]. Development and temporal cohorts were imputed separately; CT/MRI-only descriptors were treated as structurally unavailable in non-imaged encounters and were not imputed as negative findings. The outcome was included in the primary imputation model to preserve predictor–outcome associations, with complete-case and outcome-free temporal imputation analyses used as sensitivity analyses because outcome-assisted imputation can be optimistic in model evaluation [17]. The nested imputation–bootstrap approach is described by Schomaker and Heumann [18]. Detailed imputation models, pooling procedures and uncertainty limitations are provided in Supplementary Methods S4 and Table S2.

### 2.4. Model Estimation and Temporal Evaluation

M1–M3 were fitted using ridge-penalised logistic regression [19]. Continuous predictors were assessed for clinically important non-linearity with restricted cubic splines and retained as linear terms. Predictors were standardised before penalty selection, and lambda was selected in development by 10-fold cross-validation with patient clusters kept intact. The principal incremental comparison was M2 versus M1; M3, reader and sensitivity analyses were secondary or exploratory, without multiplicity adjustment. Temporal performance included AUC, Brier score, calibration intercept and slope, threshold-based operating characteristics and decision-curve analysis. Patient-level cluster bootstrap provided uncertainty for temporal performance and between-model differences (2000 resamples). A prespecified first-encounter-per-patient sensitivity analysis assessed the influence of recurrent visits. The locked continuous model specifications are reported in Supplementary Table S7. Additional fitting, sample-size-context and software details are in Supplementary Methods S4 and Tables S9 and S11.

For the mapped integer score, Brier score and calibration parameters were estimated within each of the 20 imputations and pooled using Rubin’s rules. Calibration-in-the-large used an offset with slope fixed at 1; calibration slope was estimated with a freely estimated intercept. The descriptive score frequencies in the main operating-characteristic table and Supplementary Table S12 use a single modal-category profile per encounter and are not the input used to pool these performance estimates.

### 2.5. Integer Score Construction and Clinical Utility

For score construction, continuous predictors were categorised at prespecified clinical cut-offs: age ≥ 50 years, CRP ≥ 50 mg/L, WBC ≥ 12 × 10^9^/L, temperature ≥ 38 °C and shock index ≥ 0.9. The categorical M2 model was refitted, pooled coefficients were scaled using the prespecified B = 0.40 constant and rounded as points = sign(β) × floor(|β|/0.40 + 0.5) [20]. Haematuria and hydronephrosis received zero points, producing a 12-component score ranging from −1 to 22; full coefficients and points are in Supplementary Table S6. The final point assignments are also shown in Supplementary Figure S2. The rule-out threshold was locked in development at the highest integer threshold, maintaining sensitivity ≥ 95%. This prespecified criterion was intended as a conservative safety floor for a rule-out strategy, limiting the development of a false-negative proportion among urgent events to ≤5%; it was not treated as an externally validated clinical acceptability threshold. No minimum specificity or minimum low-risk yield was prespecified. The rule-selected score was ≤−1 and was applied unchanged in temporal evaluation. Integer scores were mapped to risk using the development equation logit(p) = −3.714201 + 0.448112 × score. Following the peer-review request, a post hoc robustness analysis repeated score construction with B = 0.30 and B = 0.50, while preserving the same categorical coefficients and development threshold-selection rule; these alternatives were used only to test sensitivity to scaling and were not used to optimise the primary score.

Clinical utility was evaluated with decision-curve analysis from a 1% to 20% threshold probability [21,22]. In this framework, “treat” denotes escalation to additional urgent diagnostic or specialist management above the risk threshold, not omission of ultrasound or CT/MRI. Operating characteristics across the full attainable score range are reported in the Results; further score-construction and threshold details are provided in Supplementary Methods S4.

Local temporal calibration of the mapped B = 0.40 integer score was assessed by logistic regression with a restricted cubic spline of the logit of predicted risk. Fixed knots corresponded to probabilities 0.0368, 0.1863 and 0.7710. Predictions were averaged over the 20 imputations within each encounter before calibration fitting. The analysis included 1440 encounters from 1305 patients and 259 events; pointwise 95% confidence limits used 2000 successful patient-cluster bootstrap resamples, retaining all encounters from sampled patients. This display is distinct from calibration summaries pooled across imputations and from the modal-profile score-frequency tables.

### 2.6. Stability Analyses

Prediction and representation stability were evaluated with patient-level bootstrap resampling. Prediction-level stability and the rounding-boundary analysis used 1000 resamples. Eight hundred full-pipeline resamples repeated model fitting, penalty selection, categorical refitting, coefficient scaling and integer point assignment. A complete-card identity required every rounded component to match the development card; per-component point-change frequencies were also calculated. Classification stability required the modal low-risk/non-low-risk category to be retained in at least 95% of the 800 resamples. Detailed definitions and the full bootstrap pipeline are in Supplementary Methods S3 and S4.

## 3. Results

### 3.1. Cohort Characteristics, Incomplete Predictor Data and Outcome Distribution

The final cohort included 4800 encounters: 3360 developmental and 1440 temporal. Urgent pathology occurred in 672 development encounters (20.0%) and 259 temporal encounters (18.0%). The median age was 39.0 years (IQR 30.9–52.6). Cross-sectional imaging for the M3 information set was performed in 1152/3360 (34.3%) development encounters: CT only in 972, MRI only in 155, and both modalities in 25. In the temporal cohort, cross-sectional imaging was performed in 559/1440 (38.8%) encounters: CT only in 470, MRI only in 78, and both modalities in 11. The predominance of CT reflects the acute triage setting, particularly for suspected gastrointestinal and urological aetiologies. Mean ± SD case-mix comparisons for age, CRP, WBC, temperature and shock index showed only small period differences (absolute SMD ≤ 0.066; Supplementary Table S13).

Incomplete predictor data were unevenly distributed across periods: 40/3360 development encounters (1.2%) and 156/1440 temporal encounters (10.8%) had at least one incomplete M1/M2 predictor. Per-variable missing counts are reported in Table 1.

### 3.2. Temporal Predictive Performance

**Figure 2:**
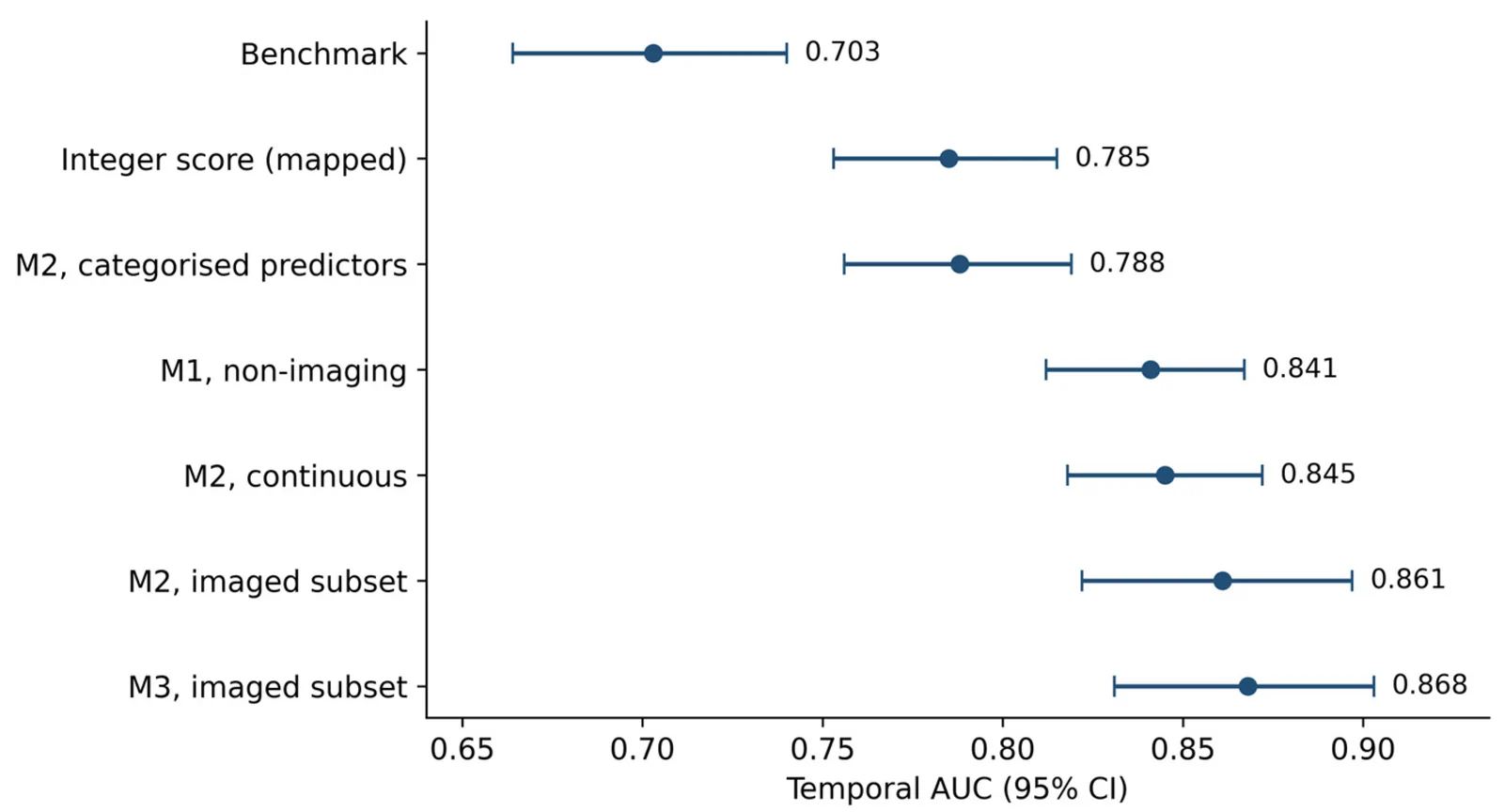
Temporal discrimination across the modelling and simplification chain, with 95% patient-cluster bootstrap intervals. The mapped integer score and the categorised-predictor model show closely similar discrimination, whereas the main loss occurs between continuous and categorised M2.

The benchmark model achieved a temporal AUC of 0.703 (95% CI 0.664–0.740), M1 0.841 (0.812–0.867) and M2 0.845 (0.818–0.872). The paired M2-versus-M1 AUC difference was 0.004 (95% patient-cluster bootstrap CI 0.001–0.009; *p* = 0.012), indicating a statistically detectable but quantitatively small gain. M2 had a Brier score of 0.104 (scaled Brier 0.295 against a reference Brier of 0.148 at the temporal prevalence of 18.0%), calibration intercept −0.178 (95% CI −0.265 to −0.091) and calibration slope 0.942 (0.851–1.033). The negative calibration intercept is compatible with a change in baseline risk and case mix between periods; the present data do not identify a unique mechanism for temporal drift. Because all included encounters had documented ultrasound, the M1-versus-M2 comparison should be interpreted as incremental predictive information from the selected ultrasound descriptors conditional on an ultrasound-documented pathway; it cannot establish whether performing ultrasound improves diagnostic triage at the pathway level or whether ultrasound can safely alter subsequent management. Temporal performance estimates are summarized in Table 2 and Figure 2; further incremental-value comparisons are reported in Supplementary Table S1.

In the prespecified sensitivity analysis restricted to the first eligible encounter per patient, the development cohort comprised 3015 encounters (618 urgent events) and the temporal cohort 1305 encounters (237 urgent events). Continuous M2 performance was materially unchanged: temporal AUC was 0.846 (95% CI 0.817–0.874), Brier score 0.103, calibration intercept −0.165 (95% CI −0.255 to −0.075), and calibration slope 0.952 (95% CI 0.855–1.049). These findings argue against substantial inflation of discrimination or calibration performance from repeated patient visits.

Reader-reproducibility analysis showed observed agreement ranging from 89.0% to 96.5% across the five ultrasound descriptors, with sample-specific Cohen’s κ ranging from 0.645 to 0.781. The reliability sample was drawn exclusively from the temporal period and comprised 192 urgent and 768 non-urgent examinations from 874 unique patients; 795 patients contributed one examination, 72 contributed two, and 7 contributed three. The outcome-stratified design therefore modestly enriched urgent examinations relative to the temporal source prevalence. When the locked M2 model was applied without refitting to the alternative ultrasound readings, discrimination remained similar: routine clinical reading AUC 0.845 (95% patient-cluster bootstrap CI 0.798–0.892), Reader 1 AUC 0.852 (0.804–0.900), and Reader 2 AUC 0.841 (0.791–0.891). The corresponding examination-level DeLong intervals were very similar (Supplementary Table S6a), indicating that explicit clustering modestly widened uncertainty without changing the substantive reader-reproducibility conclusion.

Within the 559 imaged temporal encounters (113 urgent events), M2 achieved an AUC of 0.861 and M3 0.868. The paired difference was 0.007 (95% CI −0.006 to 0.021; *p* = 0.318), providing no evidence of a clear incremental gain from the selected cross-sectional-imaging descriptors within this already-imaged subset. Under the alternative operative- or histopathology-confirmed outcome definition, 763/931 (82.0%) primary urgent events remained confirmed and 168/931 (18.0%) were excluded from the restricted event definition; in the imaged temporal subset, events decreased from 113 to 93. Among the 168 primary events without operative or histopathological confirmation, 42 were classified exclusively from ultrasound or CT/MRI reports containing descriptors also used as model predictors: 42/168 (25.0%) of unconfirmed events and 42/931 (4.5%) of all primary events. These counts document a specific source of incorporation bias; they do not establish independence of outcome assessment from predictors in the remaining events. The affected encounters were retained in the analysis denominator and coded as non-events under the restricted outcome definition. M3 carries 19 parameters; the imaged development subset contained 265 urgent events (13.9 events per parameter), compared with 48.0 for M2. Although simple events-per-parameter rules are not sufficient for prediction-model adequacy and ridge penalisation reduces overfitting, this substantially lower event support is a quantitative limitation. M3 should therefore be interpreted as an exploratory extension within a clinically selected imaged subset rather than as evidence that cross-sectional imaging should be obtained or that CT and MRI descriptors are interchangeable.

### 3.3. Simplification Loss and Rule-Out Operation Across Thresholds

**Figure 3:**
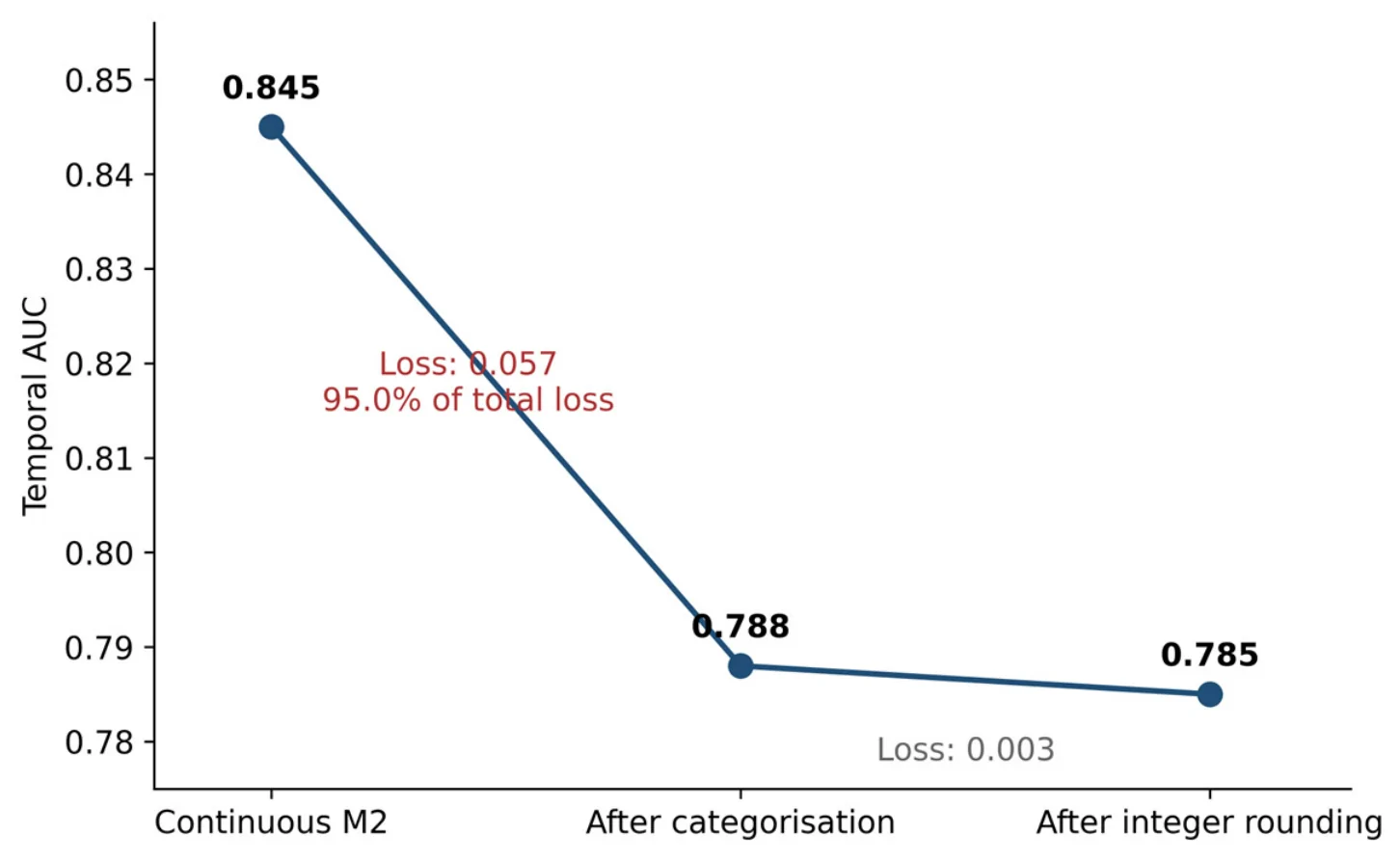
Decomposition of simplification-associated discrimination loss. Categorisation accounted for 0.057 of the total 0.060 loss; integer rounding accounted for 0.003.

Temporal AUC decreased from 0.845 for continuous M2 to 0.788 after categorisation, a loss of 0.057. Subsequent integer rounding reduced AUC by only 0.003 to 0.785. Thus 95.0% of total simplification-associated AUC loss occurred during categorisation rather than rounding. This decomposition is shown in Figure 3 and reported in detail in Supplementary Table S3.

The locked score ≤ −1 threshold classified 42 of 1440 temporal encounters (2.9%; 95% CI 2.2–3.9) as low risk. One urgent event, an atypical appendicitis, occurred in this group, yielding sensitivity 99.6% (95% CI 97.9–99.9), specificity 3.5% (2.6–4.7), negative predictive value 97.6% (87.7–99.6) and a negative likelihood ratio of 0.11 (95% CI 0.02–0.80). The wide uncertainty around the low-risk operating characteristics reflects a denominator of only 42 encounters containing a single event and should temper any interpretation of the rule as reliably exclusionary. Because −1 was the minimum attainable score, the rule selected only encounters at the floor of the score.

The adjacent score ≤ 0 threshold classified 161 of 1440 encounters (11.2%) as low risk, with sensitivity 96.9% (94.0–98.4), specificity 13.0% (11.2–15.0), negative predictive value 95.0% (90.4–97.4), LR −0.24 (0.12–0.48) and eight false negatives. A continuous M2 threshold of *p* ≤ 0.0648 classified 589 of 1440 (40.9%) as low risk, with sensitivity 93.4% (89.7–95.8), specificity 48.4% (45.6–51.3), negative predictive value 97.1% (95.4–98.2), LR −0.14 (0.09–0.22) and 17 false negatives. The two strategies are matched on the same ≥ 95% development-sensitivity criterion rather than on predicted risk; the continuous threshold can be tuned to that criterion arbitrarily finely whereas the integer score can only jump between adjacent integers. Development threshold selection and the corresponding temporal operating characteristics are summarized in Table 3; the underlying 2 × 2 counts are reported in Supplementary Table S4.

Among the 119 encounters with a score of exactly 0, seven had urgent pathology, corresponding to an observed event rate of 5.88% versus a mapped prediction of 2.38%.

**Figure 4:**
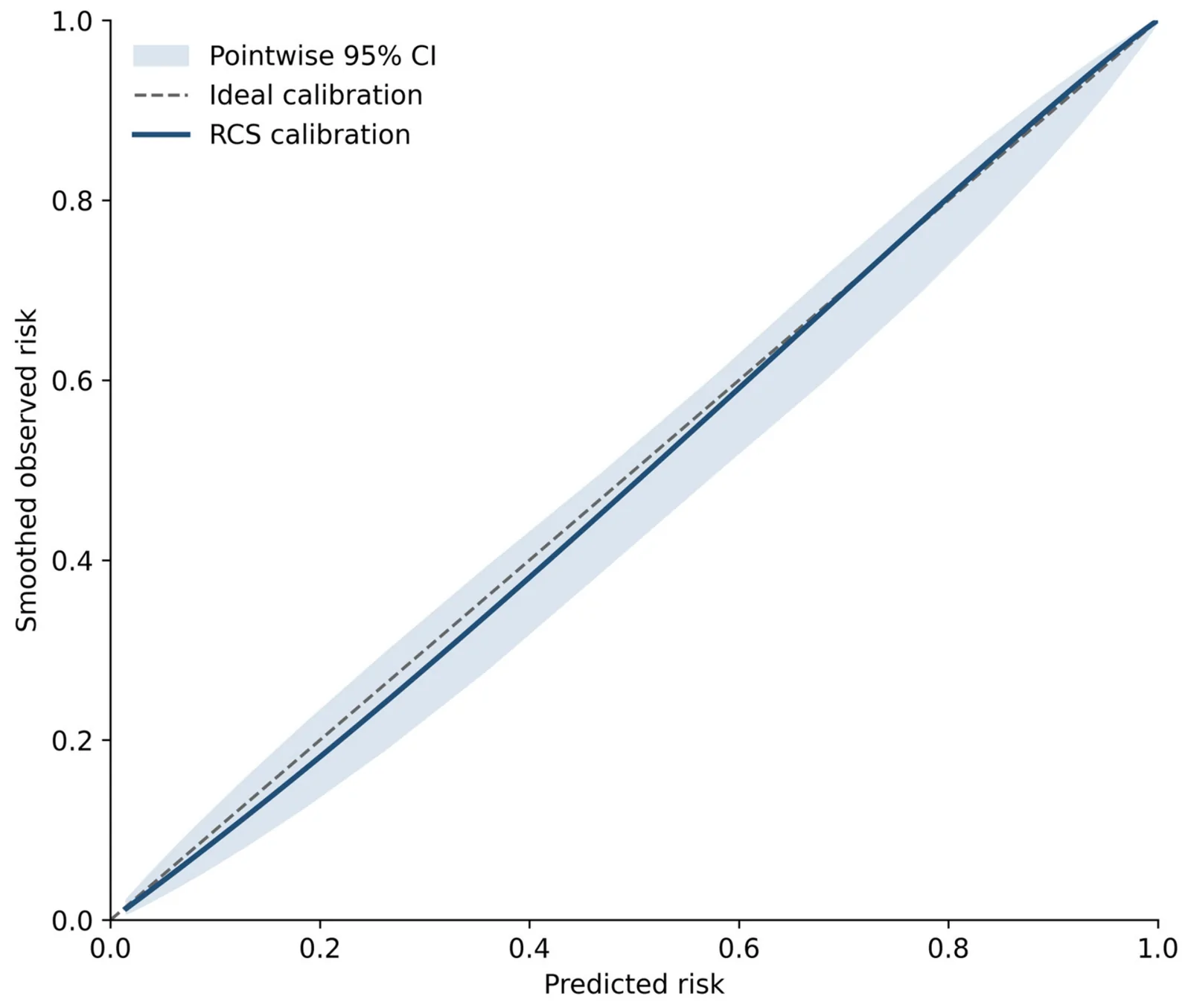
Temporal calibration of the mapped B = 0.40 integer score: 1440 encounters, 1305 patients and 259 urgent events. The solid line is a logistic restricted cubic spline fitted to the logit of encounter-level predicted probabilities averaged across 20 imputations; knots correspond to risks 0.0368, 0.1863 and 0.7710. The shaded region joins pointwise 95% confidence limits from 2000 successful patient-cluster bootstrap resamples at the supplied evaluation points. The dashed line indicates ideal calibration. The spline uses the default rms restricted-cubic-spline basis; it is separate from the descriptive modal-profile score distribution in Table 4.

The descriptive modal-profile score distribution showed an observed event rate of 5.88% at score 0 versus a mapped risk of 2.38%; at score 5, the corresponding values were 17.86% and 18.64%. The continuous calibration display based on encounter-level probabilities averaged across imputations is presented in Figure 4. This spline shows modest departures from ideal calibration and uncertainty across the prediction range. It should not be interpreted as a smoothed version of Table 4, because the two displays use different summaries of the imputations. The small low-risk group and its uncertainty remain central to clinical interpretation. The full temporal operating-characteristic profile is provided in Table 4, the development score distribution in Supplementary Table S14, and the mapped-versus-observed risk display in Supplementary Figure S1.

Development sensitivities are expressed as exact fractions of the 672 development events. Temporal sensitivity and specificity proportions carry Wilson 95% confidence intervals; negative predictive value proportions also carry Wilson 95% confidence intervals. LR− confidence intervals were calculated on the log scale from the corresponding 2 × 2 counts. Threshold selection was based on the development-sensitivity criterion; low-risk yield was not used for threshold selection. NPV, negative predictive value; FN, false negative; LR−, negative likelihood ratio. The ≥95% development-sensitivity target was a prespecified safety floor for rule-out use; no minimum specificity or low-risk yield was prespecified, which is an acknowledged design limitation.

**Figure 5:**
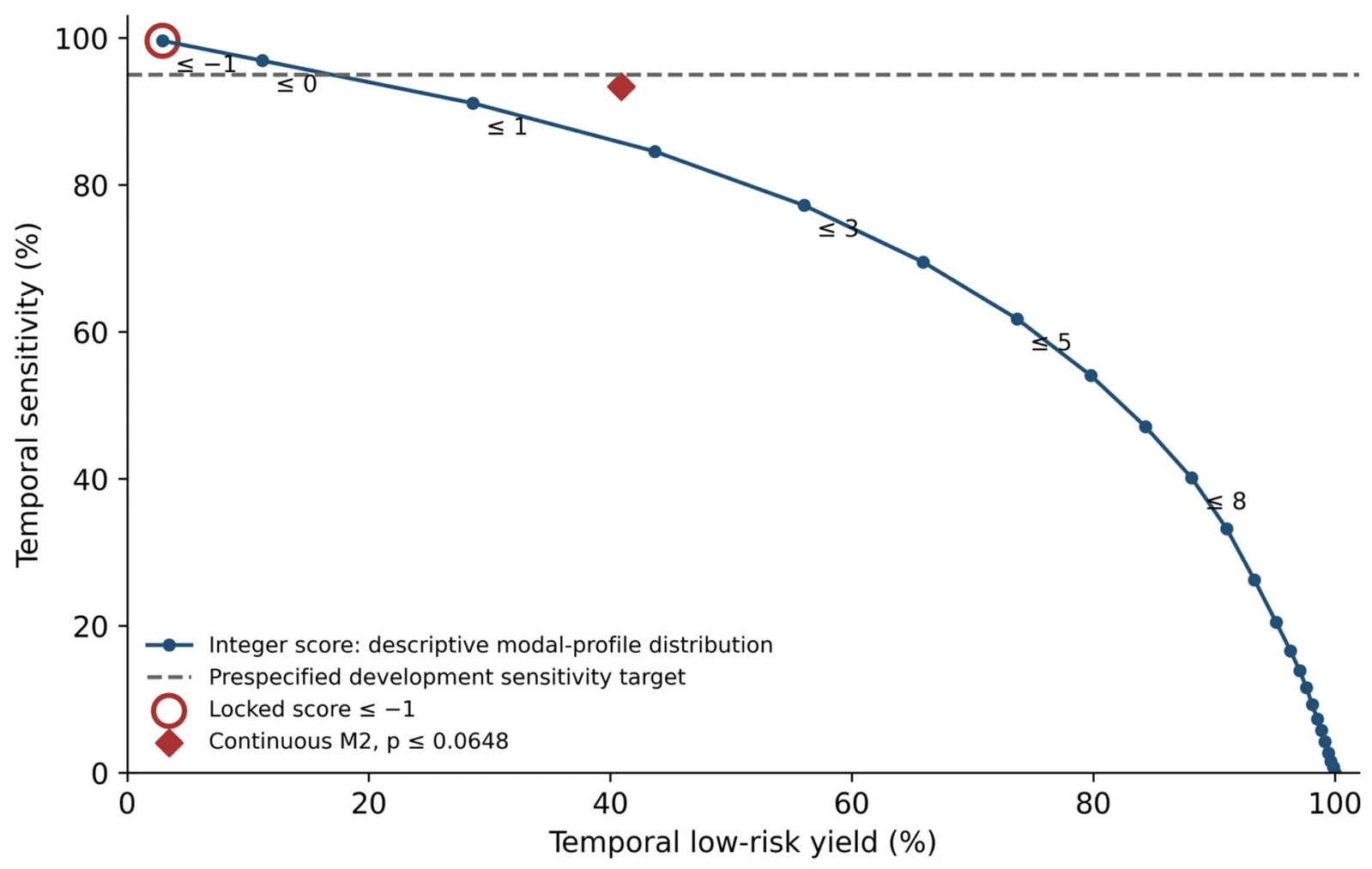
Yield–sensitivity trajectory of the integer score across attainable thresholds, with the continuous M2 comparator. The locked rule (score ≤ −1) achieved temporal sensitivity of 99.6% with a 2.9% low-risk yield; relaxing to score ≤0 increased yield to 11.2% with sensitivity of 96.9%.

Cumulative n sums to 1440 and cumulative events to 259. Observed risk rises across the score range, from 2.38% at the floor to 85.7% in the highest band. Scores above 6 are grouped because individual cells are small; the ungrouped temporal table is provided in Supplementary Table S12. The mapping underestimates risk below approximately score 5 and overestimates it above that region. At each row, “low risk” is defined as a score at or below the listed threshold; sensitivity therefore represents the proportion of urgent cases retained above the rule-out threshold. For encounters with incomplete predictors, the descriptive profile uses the modal category across the 20 imputations before the application of the fixed card. These frequencies and operating characteristics should not be equated with the pooled performance estimates in Table 2 or the mean-probability calibration display in Figure 4. The yield–sensitivity trajectory across attainable thresholds is shown in Figure 5.

Across the 1–20% decision-curve range, net benefit was 0.169 for M2 and 0.169 for M1 against 0.167 for treat-all at 1.5%; 0.139 and 0.138 against 0.123 at 6.5%; and 0.1080 and 0.1074 against 0.089 at 10%. At the previously evaluated 10% threshold the M2-minus-M1 difference was 0.0006, equivalent to approximately 0.6 additional net true-positive classifications per 1000 encounters. Here, treat-all represents escalation of all encounters rather than omission of imaging. Because the clinically relevant rule-out thresholds lie well below 10%, these selected values should be interpreted in the context of the 1–20% decision range rather than as evidence for withholding imaging.

### 3.4. Score-Card, Prediction and Classification Stability

**Figure 6:**
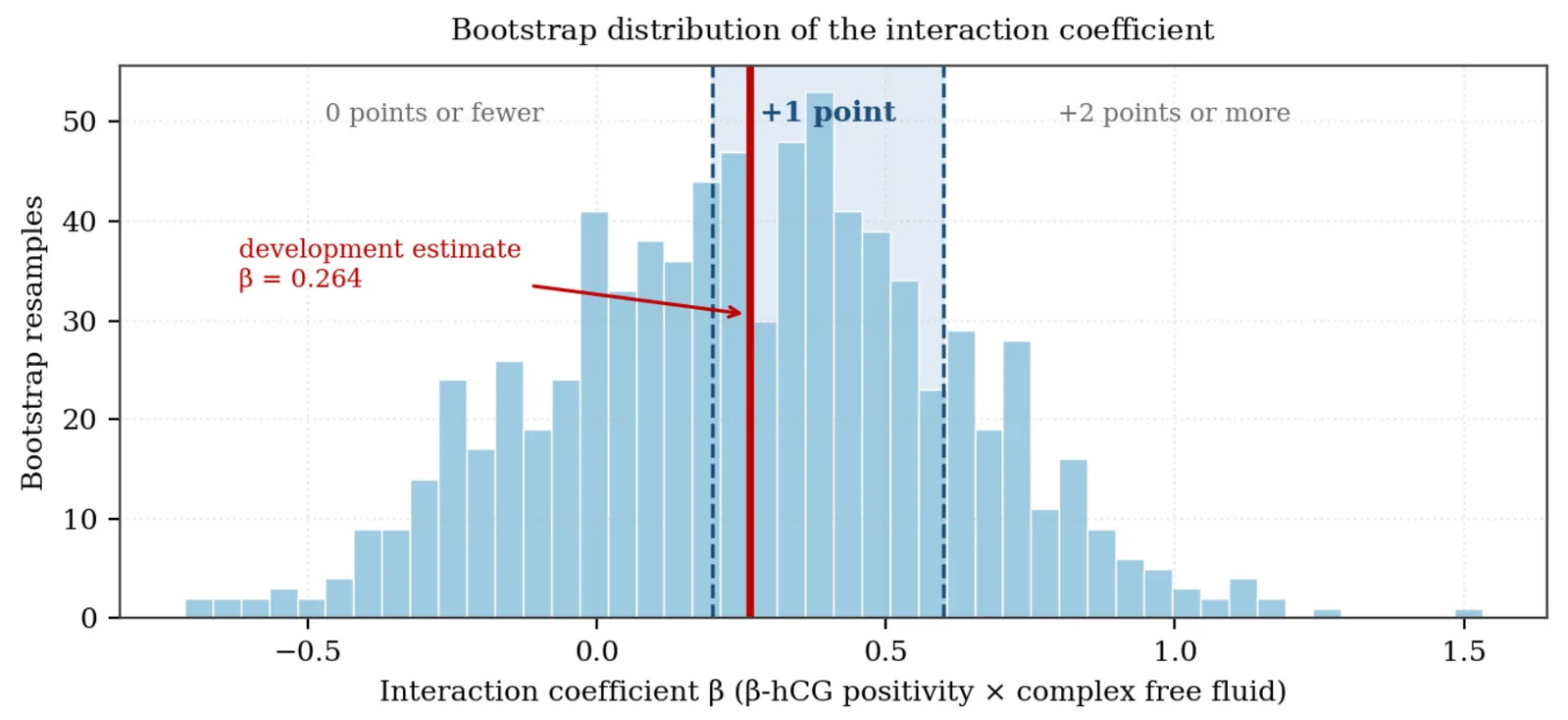
Bootstrap distribution of the interaction coefficient relative to the integer rounding boundaries. With B = 0.40, coefficients in (0.20, 0.60) receive +1 point; the development estimate of 0.264 lies 0.064 above the lower boundary.

Across 800 patient-level cluster bootstrap resamples, 798 distinct standard M2 integer score cards were generated and the original development card was reproduced once (1/800; 0.125%; 95% CI 0.003–0.694). The beta-hCG positivity × complex free-fluid interaction had a development coefficient β = 0.264, only 0.064 above the lower +1-point rounding boundary at β = 0.20. Its point assignment changed in 468/800 (58.5%) resamples. Empirical per-component coefficient standard errors, boundary distances and point-assignment change frequencies are reported in Supplementary Table S15. The bootstrap distribution of the interaction coefficient is shown in Figure 6.

In the reviewer-requested scaling-constant sensitivity analysis, the conclusion that rounding contributed little additional discrimination loss was preserved. With B = 0.30, 0.40 and 0.50, temporal integer-score AUCs were 0.786, 0.785 and 0.781, respectively, against the same categorical-M2 AUC of 0.788, corresponding to rounding losses of 0.002, 0.003 and 0.007. Score-card instability also persisted: 785, 798 and 799 unique cards were generated across 800 resamples, with exact reproduction in 4/800 (0.50%), 1/800 (0.125%) and 0/800 resamples, respectively. The interaction’s distance from its nearest rounding boundary decreased from 0.114 at B = 0.30 to 0.064 at B = 0.40 and 0.014 at B = 0.50, while its point assignment changed in 32.4%, 58.5% and 76.5% of resamples. Operational performance varied with scaling: the locked temporal low-risk yield/sensitivity was 7.5%/96.1% for B = 0.30, 2.9%/99.6% for B = 0.40 and 4.0%/98.5% for B = 0.50. Full point assignments and stability results are reported in Supplementary Tables S16 and S17.

Prediction-level variation was substantially smaller than complete-card variation. The median absolute probability difference between original and bootstrap-derived predictions was 3.4 percentage points. Using the predefined stability criterion, 96.2% of temporal encounters (1385/1440; 95% CI 95.1–97.1) remained in their modal low-risk or non-low-risk category in at least 95% of the 800 resamples. Because only 2.9% of temporal encounters were classified as low risk by the locked rule, this aggregate measure is dominated by the non-low-risk category and should not be interpreted as equivalent stability within the small low-risk subgroup.

**Figure 7:**
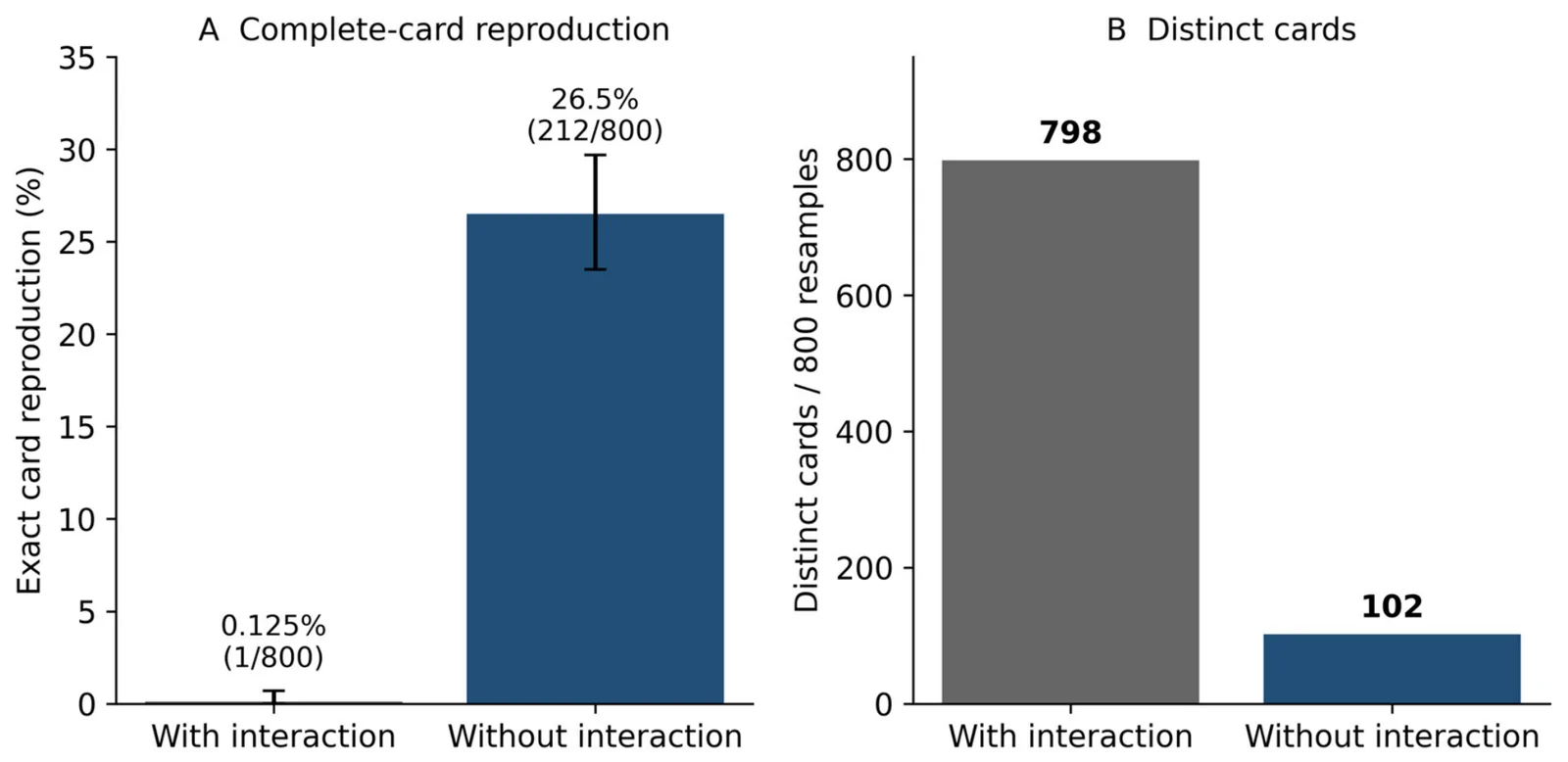
Effect of removing the sparse interaction term on score-card stability. (**A**) Exact reproducibility of the complete card; (**B**) number of distinct score cards across 800 resamples. Exact reproducibility rose more than 200-fold while temporal discrimination and rule-out performance were essentially unchanged.

In a sensitivity analysis removing the sparse interaction term, temporal discrimination remained numerically similar (AUC 0.844; 95% CI 0.816–0.871) and operational performance was virtually unchanged (sensitivity 99.6%; low-risk yield 2.8%, 40/1440). Complete-card reproducibility increased from 1/800 (0.125%; 95% CI 0.003–0.694) to 212/800 (26.5%; 95% CI 23.5–29.7), and the number of unique cards fell from 798 to 102. The interaction was retained in the primary model, and its removal was evaluated only as a stability sensitivity analysis rather than as post hoc optimisation. Detailed results are provided in Supplementary Table S5, and the corresponding changes in complete-card reproducibility and the number of distinct cards are shown in Figure 7.

### 3.5. Missing-Data and Year-Stratified Sensitivity Analyses

The complete-case temporal analysis included 1284 encounters with a complete M1/M2 predictor set, corresponding to 156 of 1440 (10.8%) temporal encounters with at least one incomplete predictor. M2 AUC was 0.846 (95% CI 0.819–0.873), Brier 0.103, calibration intercept −0.165, slope 0.950, low-risk yield 2.8% and sensitivity 99.5%. In the outcome-free temporal imputation sensitivity analysis, AUC was 0.839 (95% CI 0.811–0.865), Brier score 0.106, calibration intercept −0.192 and calibration slope 0.931. At the locked score ≤ −1 threshold, sensitivity was 99.6%, specificity 3.6%, NPV 97.5%, and low-risk yield 3.0%, and there was one false negative. Concordance across primary outcome-assisted imputation, outcome-free imputation and complete-case analysis materially reduces concern that the principal temporal conclusions are an artefact of the missing-data strategy. Because 156/1440 (10.8%) temporal encounters had at least one incomplete M1/M2 predictor and the imputation models were not re-estimated inside each bootstrap resample, the reported bootstrap confidence intervals are conditioned on the fitted imputation models and may therefore be narrower than intervals from a fully nested imputation-within-bootstrap procedure.

Encounters and urgent-event prevalence by individual calendar year are provided in Supplementary Table S10: 450/82 (18.2%) in 2013, 460/101 (22.0%), 480/91 (19.0%), 490/103 (21.0%), 500/94 (18.8%), 510/101 (19.8%) and 470/100 (21.3%) through 2019; then 390/65 (16.7%) in 2020, 520/94 (18.1%) in 2021 and 530/100 (18.9%) in 2022. These counts document that the development–temporal split followed the calendar period after removal of patient overlap.

## 4. Discussion

### 4.1. Principal Findings

The continuous M2 model maintained good discrimination in the temporal cohort, although its incremental AUC gain over the non-imaging M1 model was small. Performance was virtually unchanged when the analysis was restricted to the first eligible encounter per patient, arguing against a material contribution of recurrent visits to the reported discrimination and calibration. Most of the discrimination loss during score simplification resulted from categorisation rather than integer rounding. The resulting score also had limited rule-out yield and substantial instability of individual point assignments. Reader-specific substitution of the five ultrasound descriptors produced similar discrimination despite non-perfect agreement, supporting the robustness of the locked model to plausible reporting variation within the reliability sample. Reviewer-requested sensitivity analyses showed that the small rounding-related AUC loss and poor complete-card reproducibility persisted across B = 0.30, 0.40 and 0.50, indicating that these conclusions were not unique to the prespecified B = 0.40 representation.

Prediction-level stability was substantially greater than exact score-card reproducibility. The descriptive modal-profile score distribution also showed a discrepancy at the low end: at score 0, observed risk was 5.88% compared with a mapped risk of 2.38%. The locked score ≤ −1 rule classified only 2.9% of temporal encounters as low risk and coincided with the minimum attainable score; relaxing the threshold to score ≤0 increased yield to 11.2% but did not satisfy the prespecified development-sensitivity criterion. These findings emphasise the need to assess local calibration, operational yield and coefficient-to-point stability in the intended rule-out region.

### 4.2. Clinical Interpretation of the Tiered Pathway

The model architecture reflects progressively richer information sets rather than a fully time-stamped clinical sequence. M1 contains clinical and laboratory predictors but no imaging variables; M2 adds ultrasound findings; and M3 adds cross-sectional-imaging descriptors. M1 is not proposed as a diagnostic algorithm, a substitute for clinician assessment, or a basis for diagnosing the cause of pelvic pain without imaging. Its role in this study is as a non-imaging reference information set used to quantify the incremental predictive contribution of selected ultrasound descriptors. Because complete ultrasound documentation was required for study inclusion and retrospective timestamps did not establish a strict ordering between every M1 result, ultrasound acquisition and subsequent management decisions, the M1-versus-M2 comparison cannot estimate the pathway-level benefit of performing ultrasound, justify omission of ultrasound, or establish that ultrasound can safely alter subsequent management. If independently and prospectively validated, the continuous M2 model could at most be considered as an adjunctive post-ultrasound risk-stratification aid used alongside clinical judgement, not as an autonomous diagnostic or imaging-deferral tool.

The integer rule illustrates a structural limitation of thresholding on a coarse score: a score of ≤−1 was the minimum attainable value and could be reached only when tubal-wall thickening, the sole negatively weighted component, was present with all positively weighted components absent. An entirely unremarkable scored profile yields 0 and does not satisfy the locked rule. The negative point on tubal-wall thickening should not be read as a protective causal effect; within this multivariable model it is plausibly related to its conditional association with the non-urgent PID-without-abscess phenotype (464 encounters overall) and to redistribution of information among correlated ultrasound descriptors.

The M2-versus-M1 AUC gain of 0.004 was statistically detectable but small. The selected ultrasound descriptors therefore added limited incremental discrimination beyond the non-imaging clinical and laboratory predictors within this already ultrasound-documented modelling architecture; this is not evidence that ultrasound lacks diagnostic value or that imaging should be omitted. Because radiology reports contributed to outcome adjudication, residual incorporation bias remains possible. Of 931 primary urgent events, 763 (82.0%) had operative or histopathological confirmation and 168 (18.0%) did not; the latter were reclassified as non-events under the restricted definition, with encounters retained in the analysis denominator. Among the 168 primary events without operative or histopathological confirmation, 42 were classified exclusively from ultrasound or CT/MRI reports containing descriptors also used as model predictors: 42/168 (25.0%) of unconfirmed events and 42/931 (4.5%) of all primary events. These counts document a specific source of incorporation bias; they do not establish independence of outcome assessment from predictors in the remaining events. Within the imaged temporal subset, this restriction reduced events from 113 to 93, yet M2/M3 discrimination remained similar (AUC 0.852/0.859), which is reassuring but cannot eliminate incorporation bias because the restricted definition changes the clinical endpoint and reclassifies a substantial fraction of urgent events. Moreover, the primary endpoint was partly management-linked: the model predicts the observed combination of pathology and time-sensitive clinical management rather than an untreated latent disease state, and predictor findings may themselves have influenced clinical decisions contributing to outcome classification. M3 likewise produced only a small numerical increase within the already-imaged subset and estimates information conditional on having undergone cross-sectional imaging rather than the causal benefit of ordering it. CT predominated in both periods (972 CT-only versus 155 MRI-only development encounters, 470 versus 78 temporally), consistent with the acute triage context; therefore, the M3 results should not be interpreted as demonstrating equivalent performance of individual descriptors across CT and MRI.

The reader analysis addresses a different question from incremental-value analysis. Agreement was high but not perfect, yet reader-specific M2 AUCs remained tightly clustered around the routine-reading estimate. The sample was deliberately outcome-stratified and included repeated examinations in 79 of 874 patients. Patient-cluster bootstrap intervals were therefore used for reader AUC uncertainty and were only modestly wider than the examination-level DeLong intervals, leaving the substantive conclusion unchanged. The κ values remain sample-specific because of outcome-stratified sampling. The reader findings support robustness to plausible reporting variation in these descriptors, but they do not eliminate measurement error, incorporation bias or centre-specific reporting effects, and should not be interpreted as evidence that calibration or threshold performance will remain unchanged across readers or institutions.

### 4.3. Categorisation, Rounding and Score-Card Stability

The major loss of discrimination occurred at categorisation, not rounding, which is consistent with the well-established information loss from dichotomising continuous predictors [8]. This has practical implications because score development often focuses on the final rounding step, whereas the more consequential simplification occurred earlier. The reviewer-requested scaling sensitivity analysis reinforced this conclusion: rounding loss remained only 0.002 at B = 0.30, 0.003 at the prespecified B = 0.40 and 0.007 at B = 0.50, while exact score-card reproduction remained ≤0.50% across all three representations. Thus the qualitative instability finding is not an artefact of the single B = 0.40 choice, although the selected threshold, yield and individual point assignments remain scaling-dependent.

Complete-card reproducibility was extremely low. This criterion is intentionally stringent because all rounded weights must match simultaneously, and it is complementary to rather than interchangeable with prediction stability. The instability is nonetheless not a cosmetic artefact: the interaction lay close to a rounding boundary, and removing that sparse term improved complete-card reproducibility more than 200-fold while leaving temporal AUC, sensitivity and low-risk yield essentially unchanged. Prediction-level variation was smaller, at a median absolute difference of 3.4 percentage points, and 96.2% of encounters met the aggregate classification-stability criterion—but that percentage is driven by the non-low-risk majority, since the locked low-risk group was only 2.9% of encounters.

Per-component analysis showed the greatest point-assignment instability for the beta-hCG × complex-fluid interaction (58.5%), complex free fluid (40.2%) and positive beta-hCG (37.8%), followed by absent ovarian flow (30.1%) and WBC ≥ 12 × 10^9^/L (28.6%). These source-verified bootstrap frequencies reinforce the need to evaluate score-card stability empirically rather than assuming that a large development sample guarantees reproducible rounded weights.

### 4.4. Missing Data

Primary outcome-assisted multiple imputation, outcome-free temporal imputation and complete-case analysis produced closely similar discrimination and calibration. This three-way sensitivity analysis is important because 156/1440 temporal encounters (10.8%) had at least one incomplete M1/M2 predictor and were excluded from complete-case analysis, while outcome-assisted imputation can be optimistic and outcome-free imputation can attenuate predictor–outcome associations. Incomplete predictor data were concentrated in the temporal period, so the missing-at-random assumption bears a heavier analytical load in evaluation than in development. Separate imputation by period addresses temporal leakage but does not resolve a differential missing-data mechanism. Because imputation-model fitting was not repeated within each bootstrap resample, the reported resampling intervals are conditioned on the fitted imputation models and do not include uncertainty from refitting the imputation process; they may therefore be somewhat narrower than fully nested imputation-within-bootstrap intervals [17]. The exact degree of undercoverage cannot be quantified without rerunning that nested procedure.

### 4.5. Strengths and Limitations

Strengths include a documented prespecified SAP, the clinically staged predictor architecture, strict chronological patient-separated temporal evaluation, independent senior clinician outcome adjudication, patient-level cluster resampling, a first-encounter-per-patient sensitivity analysis, a dedicated reader-reproducibility analysis, explicit assessment of calibration and decision-analytic utility, reporting of specificity and operational yield, decomposition of simplification loss, sparse-interaction sensitivity analysis, outcome-free imputation sensitivity analysis, and the distinction between complete-card and prediction-level stability. The final continuous model specifications and integer mapping are reported in full.

Limitations remain. The retrospective single-centre design is susceptible to coding and measurement error, selection effects and unmeasured temporal changes in referral patterns, laboratory practice, imaging technology and management across the decade. The temporal window included the COVID-19 pandemic; annual counts are provided in Supplementary Table S10, but pandemic disruption cannot be separated fully from secular drift. Requiring complete ultrasound documentation for inclusion introduces selection bias; accordingly, the model target population is an ultrasound-documented diagnostic pathway rather than all-comer acute pelvic pain, and the M1-versus-M2 comparison cannot estimate the pathway-level value of performing ultrasound or establish safe imaging-deferral decisions. The primary outcome combines heterogeneous urgent diagnoses and is partly management-linked. Of 931 primary urgent events, 168 (18.0%) lacked operative/histopathological confirmation and were reclassified as non-events in the restricted-outcome analysis, with encounters retained. In 42 of these events (4.5% of all primary events), classification relied exclusively on radiological information overlapping with model predictors; other forms of incorporation bias cannot be excluded. Recurrent eligible episodes were retained as encounter-level prediction units, although the first-encounter-per-patient sensitivity analysis produced essentially unchanged M2 discrimination and calibration. The M3 analysis is restricted to a clinically selected imaged subset and contains 19 parameters for 265 development events (13.9 events per parameter), substantially less event support than M2 (48.0 per parameter); ridge penalisation mitigates but does not remove this quantitative limitation, and M3 remains exploratory. The reader analysis involved only two alternative readers and an outcome-stratified sample, but patient-cluster bootstrap AUC intervals accounting for the 79 patients with repeated examinations were similar to the original examination-level intervals. Outcome-assisted imputation is not a real-time solution for missing predictors; 156/1440 temporal encounters (10.8%) had incomplete M1/M2 predictor data, and because the imputation model was not refitted within every bootstrap resample, uncertainty intervals may be somewhat narrow. The prespecified ≥95% development-sensitivity criterion was a conservative safety floor rather than a validated clinical threshold, and no minimum specificity or clinically meaningful low-risk yield was prespecified; the resulting 2.9% temporal yield demonstrates why both should be considered in future prospective rule design. Several key operating characteristics, notably the negative predictive value of 97.6%, rest on a very small low-risk denominator and therefore have wide uncertainty. The discrepancy at score 0 in the descriptive model-profile distribution, the small low-risk denominator and the distinct handling of imputations in the performance and calibration displays limit direct risk communication from the integer card. Post hoc scaling sensitivities at B = 0.30 and B = 0.50 showed that low rounding loss and poor card reproducibility were not unique to B = 0.40, but they do not establish an optimal scaling constant. Finally, temporal evaluation at a single centre does not establish transportability across institutions, prevalence settings or clinical pathways; independent multicentre evaluation and prospective workflow assessment are required before implementation. No model or integer score should be used autonomously to defer imaging, admission, specialist evaluation or intervention on the basis of these data alone. Additional interpretation boundaries are provided in Supplementary Note S1.

## 5. Conclusions

In this retrospective cohort with strict patient-separated temporal evaluation, the continuous M2 model showed good discrimination but only a small incremental improvement over the non-imaging M1 model. Because all included encounters had documented ultrasound, this comparison quantifies incremental model information within an ultrasound-documented pathway and does not establish the pathway-level diagnostic benefit of ultrasound or the safety of changing subsequent management. Simplification to an integer score reduced discrimination primarily through categorisation rather than rounding, and exact score-card reproducibility was very low across bootstrap resamples despite substantially better prediction-level stability. The descriptive modal-profile distribution showed a low-score observed-versus-mapped risk discrepancy, and the integer rule had limited rule-out yield.

These findings favour retention of the continuous model for further independent evaluation and show that any simplified rule should undergo additional assessment of local calibration, operational yield and coefficient-to-point stability before clinical implementation. The persistence of these findings across B = 0.30, 0.40 and 0.50 supports their robustness to reasonable changes in the scaling constant, but does not identify an optimal integer representation.

## Figures and Tables

**Table 1:** Baseline characteristics of the development and temporal-evaluation cohorts.

Characteristic	Overall (*N* = 4800)	Development (*n* = 3360)	Temporal (*n* = 1440)	Missing, Dev	Missing, Temp
Age, y, median (IQR)	39.0 (30.9–52.6)	38.7 (30.7–52.1)	39.7 (31.3–54.2)	0	0
CRP, mg/L, median (IQR)	7.2 (0.0–46.0)	7.8 (0.0–46.7)	5.5 (0.0–44.4)	8	34
WBC, ×10^9^/L, median (IQR)	8.6 (6.5–10.9)	8.7 (6.4–11.0)	8.5 (6.5–10.7)	4	14
Temperature, °C, median (IQR)	36.9 (36.5–37.3)	36.9 (36.5–37.3)	36.9 (36.5–37.3)	3	9
Shock index, median (IQR)	0.6 (0.6–0.7)	0.6 (0.6–0.7)	0.6 (0.6–0.7)	1	4
Positive beta-hCG, n (%)	247 (5.1)	156 (4.6)	91 (6.3)	0	0
Peritonism, n (%)	960 (20.0)	675 (20.1)	285 (19.8)	3	11
Adnexal mass, n (%)	862 (18.0)	609 (18.1)	253 (17.6)	7	28
Complex free fluid, n (%)	884 (18.4)	611 (18.2)	273 (19.0)	7	28
Absent ovarian flow, n (%)	344 (7.2)	234 (7.0)	110 (7.6)	7	28
Tubal-wall thickening, n (%)	558 (11.6)	395 (11.8)	163 (11.3)	7	28
Hydronephrosis on US, n (%)	803 (16.7)	579 (17.2)	224 (15.6)	7	28
Haematuria, n (%)	929 (19.4)	671 (20.0)	258 (17.9)	18	70
CT/MRI performed, n (%)	1711 (35.6)	1152 (34.3)	559 (38.8)	0	0
≥1 incomplete M1/M2 predictor, n (%)	196 (4.1)	40 (1.2)	156 (10.8)	—	—
Urgent pathology, n (%)	931 (19.4)	672 (20.0)	259 (18.0)	0	0

Values are n (%) unless otherwise indicated; *N* = 4800 overall, *n* = 3360 development and *n* = 1440 temporal. Missing values are reported separately. Pooled missing counts in the source data were CRP 42, WBC 18, temperature 12, shock index 5, peritonism 14, haematuria 88, and 35 for each of the five ultrasound descriptors; the development/temporal columns provide the period-specific split. CRP, C-reactive protein; WBC, white blood cell count; US, ultrasound. No hypothesis tests were performed for baseline period comparisons; differences were described using standardised mean differences, with continuous-variable SMDs reported in Supplementary Table S13.

**Table 2:** Predictive performance in the temporal-evaluation cohort.

Model	*N*	Events	AUC (95% CI)	Brier	Cal. Intercept (95% CI)	Cal. Slope (95% CI)
Benchmark	1440	259	0.703 (0.664–0.740)	0.133	−0.204 (−0.312 to −0.096)	0.950 (0.842–1.058)
M1	1440	259	0.841 (0.812–0.867)	0.106	−0.212 (−0.301 to −0.123)	0.915 (0.820–1.010)
M2 (continuous)	1440	259	0.845 (0.818–0.872)	0.104	−0.178 (−0.265 to −0.091)	0.942 (0.851–1.033)
M2, categorised predictors, continuous coefficients	1440	259	0.788 (0.756–0.819)	0.115	−0.118 (−0.194 to −0.042)	0.987 (0.902–1.072)
Integer score (mapped)	1440	259	0.785 (0.753–0.815)	0.118	−0.109 (−0.198 to −0.020)	1.013 (0.910–1.116)
M2, imaged subset	559	113	0.861 (0.822–0.897)	0.110	−0.250 (−0.380 to −0.120)	0.964 (0.830–1.098)
M3, same imaged subset	559	113	0.868 (0.831–0.903)	0.105	−0.107 (−0.231 to 0.017)	1.114 (0.970–1.258)
M2/M3, operative- or histopathology-confirmed outcome definition	559	93	0.852/0.859	—	—	—

**Table 3:** Development threshold selection and temporal rule-out operating characteristics.

Strategy	Threshold	Development Sensitivity	Temporal Sensitivity (95% CI)	Temporal Spec./LR−	Temporal NPV (95% CI); Yield; FN
Locked integer score	Score ≤ −1	99.4% (668/672)	99.6% (97.9–99.9)	3.5% (2.6–4.7)/0.11 (0.02–0.80)	97.6% (87.7–99.6); 2.9%; 1 FN
Adjacent integer	Score ≤ 0	94.0% (632/672)	96.9% (94.0–98.4)	13.0% (11.2–15.0)/0.24 (0.12–0.48)	95.0% (90.4–97.4); 11.2%; 8 FN
Continuous M2	*p* ≤ 0.0648	95.1% (639/672)	93.4% (89.7–95.8)	48.4% (45.6–51.3)/0.14 (0.09–0.22)	97.1% (95.4–98.2); 40.9%; 17 FN

**Table 4:** Full temporal operating-characteristic profile of the integer score.

Score	n	Events	Observed Risk	Mapped Risk	Cum. n (%)	Cum. FN	Sens.	Spec.	NPV	LR−
−1	42	1	2.38%	1.53%	42 (2.9)	1	99.6%	3.5%	97.6%	0.11
0	119	7	5.88%	2.38%	161 (11.2)	8	96.9%	13.0%	95.0%	0.24
1	251	15	5.98%	3.68%	412 (28.6)	23	91.1%	32.9%	94.4%	0.27
2	217	17	7.83%	5.64%	629 (43.7)	40	84.6%	49.9%	93.6%	0.31
3	178	19	10.67%	8.55%	807 (56.0)	59	77.2%	63.3%	92.7%	0.36
4	142	20	14.08%	12.77%	949 (65.9)	79	69.5%	73.7%	91.7%	0.41
5	112	20	17.86%	18.64%	1061 (73.7)	99	61.8%	81.5%	90.7%	0.47
6	88	20	22.73%	26.40%	1149 (79.8)	119	54.1%	87.2%	89.6%	0.53
7–9	162	54	33.3%	36.0–57.9%	1311 (91.0)	173	33.2%	96.4%	86.8%	0.69
10–13	87	50	57.5%	68.3–89.2%	1398 (97.1)	223	13.9%	99.5%	84.0%	0.87
14–22	42	36	85.7%	92.8–99.8%	1440 (100.0)	259	0.0%	100.0%	82.0%	1.00

## Data Availability

The analysis code presented in this article is not readily available because it is retained in a secure institutional repository and is subject to institutional governance requirements. Requests to access the analysis code should be directed to the corresponding authors.

## Supplementary Materials

The following supporting information can be downloaded at: https://www.mdpi.com/article/10.3390/diagnostics16183003/s1, Supplementary Materials include Supplementary Methods S1–S4 and S2a–S2b; Tables S1–S17 including Table S6a; Supplementary Note S1; Figure S1: Mapped versus observed risk by integer score in the descriptive modal-profile temporal distribution (Table S12). Points show observed event rates with 95% Wilson confidence intervals; the fitted development score-to-risk mapping is shown as a continuous curve. The largest clinically relevant discrepancy at the rule-out end occurs at score 0 (7/119 events; 5.88% observed versus 2.38% mapped); Figure S2: Final categorical M2 integer point assignments under B = 0.40. Eleven components carry positive weight, two carry zero weight and one carries −1; Table S1: Incremental-value comparisons; Table S2: Missing-data analysis strategies, temporal cohort; Table S3: Decomposition of simplification loss; Table S4: Counts underlying temporal specificity and negative likelihood ratios; Table S5: Sensitivity analysis removing the sparse interaction term; Table S6: Final development score card and calibration parameters; Table S6a: Inter-reader reproducibility and reader-specific model discrimination; Table S7: Locked continuous model specifications; Table S8: Mutually exclusive clinical diagnoses and outcome mapping; Table S9: Post-hoc sample-size context; Table S10: Encounters and urgent events by calendar year; Table S11: Penalty selection and single-penalty sensitivity analysis; Table S12: Ungrouped temporal operating profile of the integer score; Table S13: Development–temporal case-mix comparison using mean ± SD and standardized mean differences; Table S14: Development distribution of the integer score; Table S15: Empirical coefficient uncertainty and integer point-assignment stability; Table S16: Sensitivity of integer-score performance and stability to the scaling constant B; Table S17: Point assignments and bootstrap point-change frequencies across scaling constants; Methods S1: Cohort definition and data extraction; Method S2: Operational predictor definitions; Methods S2a: Operational definitions of urgent outcome categories; Methods S2b: Reader-reproducibility analysis; Methods S3: Bootstrap stability pipeline; Methods S4: Additional statistical, imputation and score-construction details; Note S1. Interpretation boundaries.
