# Supplementary material for: Development and Temporal Evaluation of a Tiered Clinical–Ultrasound Prediction Model and Simplified Integer Score for Time-Sensitive Pathology in Women with Acute Pelvic Pain: A Retrospective Cohort Study
Source: Diagnostics (Basel). 2026 Sep 16;16(18):3003. doi: 10.3390/diagnostics16183003 (PMC13604951; doi:10.3390/diagnostics16183003)
Supplement: Supplementary file 1 [file diagnostics-16-03003-s001.zip › diagnostics-4522495-supplementary.pdf]

Supplementary Materials

Development and Temporal Evaluation of a Tiered Clinical–Ultrasound Prediction Model and Simplified Integer Score for Time-Sensitive Pathology in Women With Acute Pelvic Pain: A Retrospective Cohort Study

Marijana Basta Nikolić, Dragan Vasin, Jelena Pilipović Grubor, Mirela Juković, Dijana Nićiforović, Dragan Nikolić, Sanja Stojanović

Contents. Supplementary Methods S1–S4 and S2a–S2b; Tables S1–S17 including Table S6a; Supplementary Note S1; Figures S1–S2. TRIPOD+AI and STROBE reporting checklists are supplied as separate files.

Supplementary Methods S1. Cohort definition and data extraction

Initial electronic health record screening identified 7,862 encounters. After excluding age <18 years (n = 610), trauma or postoperative pain within 30 days (n = 430), active pelvic malignancy (n = 120), pregnancy beyond 20 weeks (n = 340), missing primary outcome (n = 310), incomplete ultrasound documentation (n = 610) and duplicate visits within the same 30-day clinical episode (n = 430), 5,012 eligible encounters remained. Encounters from 2020–2022 belonging to patients already included during 2013–2019 (212 encounters from 184 patients) were removed from temporal evaluation, leaving 4,800 analysed encounters in 4,320 unique patients. Across the whole cohort, 196 encounters (4.1%) had at least one incomplete M1/M2 predictor: 40 in the development period (1.2%) and 156 in the temporal period (10.8%). All extraction was performed after ethics approval was granted on 15 January 2025. Because complete ultrasound documentation was required for inclusion, the resulting model target population is an ultrasound-documented acute-pelvic-pain diagnostic pathway rather than all unselected emergency presentations. The unit of prediction was the encounter; recurrent eligible episodes were retained, with patient clustering preserved in cross-validation and bootstrap procedures. In a prespecified first-encounter-per-patient sensitivity analysis, 3,015 development encounters (618 urgent events) and 1,305 temporal encounters (237 urgent events) remained.

Among the 168 primary events without operative or histopathological confirmation, 42 were classified exclusively from ultrasound or CT/MRI reports containing descriptors also used as model predictors: 42/168 (25.0%) of unconfirmed events and 42/931 (4.5%) of all primary events. These counts document a specific source of incorporation bias; they do not establish independence of outcome assessment from predictors in the remaining events. In the restricted-outcome sensitivity analysis, all 168 unconfirmed primary events were reclassified as non-events while their encounters remained in the denominator.

Supplementary Methods S2. Operational predictor definitions

- Positive beta-hCG: serum beta-hCG >5 IU/L or a positive qualitative urine beta-hCG test at index presentation.
- Haematuria: dipstick ≥1+ or microscopic confirmation (>3 red blood cells per high-power field).
- Peritonism: documented rebound tenderness, rigidity or guarding on physical examination.
- Adnexal mass: any solid, cystic or complex ovarian or para-ovarian lesion ≥3 cm in maximum diameter, explicitly excluding simple anechoic physiological follicles.
- Complex free fluid: echogenic or loculated pelvic free fluid on ultrasound.
- Absent or diminished ovarian flow: asymmetrical lack or marked reduction of venous or arterial spectral Doppler flow compared with the contralateral ovary.
- Tubal-wall thickening: fallopian-tube wall thickness ≥5 mm on ultrasound.
- Hydronephrosis on ultrasound: moderate-to-severe pelvicalyceal dilatation with clear calyceal involvement, with or without cortical thinning.
- Shock index: heart rate divided by systolic blood pressure, from the first recorded paired vital signs obtained at emergency triage within 60 minutes of registration.
- CRP, WBC and temperature: from the initial blood draw and triage assessment, with collection or measurement within 4 hours of index presentation.

- Perinephric stranding: reported inflammatory stranding or oedematous change in the perirenal fat on cross-sectional imaging.
- Bowel-wall discontinuity: focal interruption or frank anatomical disruption of the bowel-wall contour; reduced or absent mural enhancement alone was not counted.
- Mesenteric fat stranding: reported inflammatory stranding or oedematous change in mesenteric fat on cross-sectional imaging.
- Free extraluminal gas or fluid: reported ectopic gas or non-physiological extraluminal fluid outside expected physiological spaces, unexplained by recent surgical intervention. Cross-sectional-imaging descriptors were abstracted from formal CT or MRI reports using modality-appropriate wording; CT-specific attenuation terminology was not required for MRI examinations. Among development M3 encounters, 972 had CT only, 155 MRI only and 25 both modalities (total 1,152); among temporal M3 encounters, 470 had CT only, 78 MRI only and 11 both modalities (total 559).

Supplementary Methods S2a. Operational definitions of urgent outcome categories

Each category required an adjudicated final diagnosis meeting the primary urgent-outcome criterion of time-sensitive therapeutic intervention or specialised inpatient management within 72 hours of presentation. “Specialised inpatient management” required inpatient management by the relevant specialty for the adjudicated urgent diagnosis within the prespecified window; routine observation alone, conservatively managed presentations and interventions outside 72 hours did not qualify. Operationally, the urgent categories were: adjudicated adnexal torsion requiring therapeutic intervention; appendicitis requiring urgent management; bowel obstruction, perforation or ischaemia requiring therapeutic intervention or specialised inpatient management; ectopic pregnancy requiring intervention; infected obstructed urinary tract pathology requiring urgent inpatient treatment or drainage; and the prespecified vascular emergencies described below. Adjudication was performed independently by two senior attending physicians (a gynaecologist and a general surgeon, each with >15 years of acute-care experience). Disagreements occurred in 42/931 (4.5%) urgent cases and were resolved by a third senior adjudicator, a subspecialist in emergency radiology. Of the 931 adjudicated urgent events, 763 (82.0%) were confirmed by operative findings or histopathology rather than by imaging alone. In the alternative-outcome sensitivity analysis, imaging-only urgent cases remained in the denominator but did not meet the event definition; they were not excluded from the cohort.

The 54 adjudicated vascular emergencies comprised ovarian vein thrombosis (n = 39), extensive pelvic thrombophlebitis or deep pelvic venous thrombosis (n = 9), and spontaneous rupture of pelvic vessels or bleeding pelvic arteriovenous malformation (n = 6). These three subcategories are nested within the 54 and are not additive to it.

Severe pelvic inflammatory disease requiring surgical drainage, and haemodynamically significant haemorrhagic ovarian cyst requiring emergency surgery, were prespecified intervention-linked urgent categories but had zero final adjudicated events. Under the study definition, conservatively managed cases and interventions occurring outside the prespecified 72-hour window were not counted in these categories. The zero counts reflect the narrow time-sensitive endpoint and do not imply absence of these presentations from the source population.

Supplementary Methods S2b. Reader-reproducibility analysis

An outcome-stratified random sample of 960 ultrasound examinations was drawn exclusively from the temporal-evaluation period: 192 urgent and 768 non-urgent examinations, corresponding to sampling fractions of 74.1% (192/259) and 65.0% (768/1,181), respectively. The examinations belonged to 874 unique patients: 795 patients contributed one examination, 72 contributed two, and 7 contributed three (maximum 3). The examinations were independently and retrospectively reviewed by two radiologists: Reader 1, a senior attending radiologist with >15 years of experience in emergency and urogenital imaging, and Reader 2, a board-certified radiologist with 6 years of post-residency experience. The readers evaluated the original archived static images and cine loops stored in PACS and were strictly blinded to the final adjudicated clinical outcome, the original routine clinical ultrasound report, the patient's surgical and electronic health records, and each other's readings. The paired binary classifications were retained for complex free fluid, absent or diminished ovarian flow, adnexal mass, tubal-wall thickening and hydronephrosis. Observed agreement and Cohen's kappa were calculated directly from the original 2×2 tables. Because the sample was outcome-stratified, these agreement statistics are interpreted as reliability-sample estimates; no prevalence reweighting was applied. For reader-level discrimination, each reader's ultrasound

classifications were substituted into the locked development M2 model without coefficient refitting. Because 79 of 874 patients contributed repeated examinations, reader-specific 95% AUC CIs were obtained by patient-cluster bootstrap, resampling unique patients so that all examinations from a sampled patient remained within the cluster. Original examination-level DeLong CIs were retained as a descriptive sensitivity comparison in Table S6a. No reader-specific threshold was selected and the reader substudy was not used for model recalibration.

Supplementary Methods S3. Bootstrap stability pipeline

Patient identifiers were sampled with replacement and all eligible encounters belonging to a sampled patient were retained. For the 800 full-pipeline score-card resamples, model fitting, penalty selection, categorical refitting, coefficient scaling and integer point assignment were repeated in full. Complete-card identity required all rounded predictor weights to match the development card simultaneously and is therefore an intentionally stringent measure, complementary to prediction-level stability. The empirical coefficient distribution from these 800 resamples was used to calculate per-component bootstrap standard errors and point-assignment change frequencies (Table S15). Prediction-level stability and the dedicated rounding-boundary analysis used 1,000 patient-cluster resamples. Classification stability required the modal low-risk or non-low-risk category to be retained in at least 95% of 800 full-pipeline resamples, consistent with Section 2.6 of the main text.

Supplementary Methods S4. Additional statistical, imputation and score-construction details
Missing-data procedure. Multiple imputation by chained equations (MICE) used 20 imputed datasets and 20 iterations per dataset. Convergence was assessed from trace plots of chain means and standard deviations. Continuous variables were imputed by predictive mean matching and binary variables by logistic regression. The imputation model included all analysis predictors, the outcome, and prespecified auxiliary variables (heart rate, systolic and diastolic blood pressure, respiratory rate, parity, prior abdominal or pelvic surgery, pain score, and symptom duration). Development and temporal cohorts were imputed separately. Ridge models were fitted within each imputed dataset; coefficients were combined at parameter level using Rubin's rules. Because separately tuned penalised models do not have an exact Rubin-rule analogue, a single-penalty sensitivity analysis is reported in Table S11. Predictive-performance uncertainty was obtained from patient-cluster bootstrap across the multiply imputed analyses; imputation itself was not repeated within every bootstrap resample, so intervals may under-represent imputation-model uncertainty [18]. For the descriptive integer-score frequency distribution, incomplete categorical predictors were represented by the modal category across 20 imputations for each encounter; the single pooled-coefficient card was then applied to that profile. This was an analytical evaluation device and is not proposed as a real-time missing-data strategy. The temporal complete-case M1/M2 sample was n = 1,284, meaning that 156/1,440 (10.8%) temporal encounters had at least one incomplete M1/M2 predictor. Because imputation was not repeated inside every bootstrap resample, the reported bootstrap intervals condition on the fitted imputation models and may be narrower than intervals from a fully nested imputation-within-bootstrap procedure; the magnitude of this undercoverage cannot be quantified without rerunning that nested procedure.
For the mapped integer score, Brier score and calibration parameters were estimated within each of the 20 imputations and pooled using Rubin's rules. Calibration-in-the-large used an offset with slope fixed at 1; calibration slope was estimated with a freely estimated intercept. The descriptive score frequencies in Table 4 and Table S12 use a single modal-category profile per encounter and are not the input used to pool these performance estimates. Local temporal calibration of the mapped B = 0.40 integer score was assessed by logistic regression with a restricted cubic spline of the logit of predicted risk. Fixed knots corresponded to probabilities 0.0368, 0.1863 and 0.7710. Predictions were averaged over the 20 imputations within each encounter before calibration fitting. The analysis included 1,440 encounters from 1,305 patients and 259 events; pointwise 95% confidence limits used 2,000 successful patient-cluster bootstrap resamples, retaining all encounters from sampled patients. This display is distinct from calibration summaries pooled across imputations and from the modal-profile score-frequency tables.
Model fitting and evaluation. Continuous predictors were examined with restricted cubic splines using three knots; no departures judged clinically important enough to justify a more complex functional form were retained. Continuous and binary predictors were standardised before ridge penalisation, with the intercept unpenalised; the prediction equations must be evaluated using the explicit predictor coding in Table S7. Lambda was selected by minimum 10-fold cross-validated deviance with patient clusters kept intact within folds (development $\lambda = 0.019$ for M1, 0.023 for M2 and 0.035 for M3; M2 inter-imputation range 0.018–0.028). Patient-cluster bootstrap rather than standard DeLong inference was used for the primary M2-versus-M1 AUC difference because repeated encounters could violate independence. Temporal performance confidence intervals and AUC differences used 2,000 resamples; prediction/boundary analyses used 1,000; complete-card and component-level score stability used 800 full-pipeline resamples. A first-encounter-per-patient sensitivity analysis repeated M2 estimation and temporal evaluation in independent patient-level samples. The post-hoc sample-size context in Table S9 used 14 candidate parameters, development prevalence 0.20 and apparent Cox–Snell $R^2 = 0.276$; it was descriptive rather than a prospective design calculation.

Integer score and clinical utility. Prespecified cut-offs were age ≥50 years, CRP ≥50 mg/L, WBC ≥12 × 10^9/L, temperature ≥38 °C and shock index ≥0.9. After categorical refitting, pooled back-transformed coefficients were divided by B = 0.40 and rounded as sign(β) × floor(|β|/0.40 + 0.5). B = 0.40 was fixed in the SAP and was not selected by searching model performance. The primary integer rule was the highest development threshold maintaining sensitivity ≥95%; this prespecified target was intended as a conservative safety floor for rule-out use, limiting the development false-negative proportion among urgent events to ≤5%. It was not an externally validated clinical acceptability threshold. No minimum specificity or low-risk yield was prespecified. Score ≤−1 met the primary rule, whereas score ≤0 did not; the threshold was not re-selected temporally. Score probabilities were obtained from logit(p) = −3.714201 + 0.448112 × score. Following peer-review request, post hoc sensitivity analyses repeated point scaling with B = 0.30 and B = 0.50 using the same categorical coefficients and the same development threshold-selection rule. These analyses were performed to test robustness to scaling and were not used to choose or optimise the primary B = 0.40 score. Decision-curve analysis evaluated threshold probabilities from 1% to 20%; "treat" represented escalation to further urgent diagnostic/specialist management above the risk threshold. Prediction-level stability was the absolute difference between the locked-model probability and each bootstrap-derived probability; complete-card identity required all rounded predictor weights to match simultaneously. Classification stability was defined as retention of the modal low-risk/non-low-risk category in at least 95% of 800 full-pipeline resamples.

Software and reproducibility. Analyses were performed in R version 4.3.1 (R Foundation for Statistical Computing, Vienna, Austria), including mice version 3.16.0, glmnet version 4.1-8 and pmsampsize version 1.1.3. The analysis code is retained in a secure institutional repository and is available from the corresponding author on reasonable request, subject to institutional governance requirements.

Main Figure 4 uses logistic restricted cubic splines on logit(predicted risk), with default rms normalisation (norm = 2). The final calibration coefficients are intercept −0.121293841, linear term 1.009775201 and nonlinear term 0.041829315; knots are logit(0.0368), logit(0.1863) and logit(0.7710). These coefficients describe the calibration display, not a replacement prediction model or the pooled global calibration slope. The pointwise bootstrap limits are shown at the supplied evaluation grid; the central curve is evaluated directly from these coefficients.

Supplementary Table S1. Incremental-value comparisons

Comparison	Δ AUC	95% cluster-bootstrap CI	Bootstrap P	Δ Brier	Δ net benefit at 0.10
M2 vs M1, all temporal	0.004	0.001 to 0.009	0.012	0.002	+0.0006
M3 vs M2, same imaged subset	0.007	−0.006 to 0.021	0.318	0.005	—
M2 vs benchmark	0.142	0.115 to 0.169	<0.001	0.029	—
M2 continuous vs categorised	0.057	—	—	0.011	—
Categorised vs integer score	0.003	—	—	0.003	—

Brier differences are arithmetic differences between the three-decimal Brier scores in main Table 2. Net benefit at a 10% threshold probability is reported for the principal comparison only; the full decision curve from 1% to 20% is described in Section 3.3 of the main text.

Supplementary Table S2. Missing-data analysis strategies, temporal cohort

Analysis	N	Events	Coverage	AUC (95% CI)	Brier	Cal. intercept	Cal. slope	Yield / sens.
Multiple imputation (m = 20)	1,440	259	100%	0.845 (0.818–0.872)	0.104	−0.178	0.942	2.9% / 99.6%
Complete case	1,284	231	89.2%	0.846 (0.819–0.873)	0.103	−0.165	0.950	2.8% / 99.5%
Outcome-free imputation	1,440	259	100%	0.839 (0.811–0.865)	0.106	−0.192	0.931	3.0% / 99.6%

The complete-case denominator is defined strictly by availability of the complete M1/M2 clinical, laboratory and ultrasound predictor set, independently of whether CT/MRI was performed. Development complete-case coverage was 98.8% against 89.2% temporally. In the outcome-free temporal imputation analysis, the locked score ≤−1 threshold had sensitivity 99.6%, specificity 3.6%, NPV 97.5%, low-risk yield 3.0% and 1 false negative. The three approaches support the same substantive conclusion despite different assumptions about missing predictors.

First-encounter-per-patient sensitivity analysis. Restricting the data to the first eligible encounter for each patient yielded 3,015 development encounters with 618 urgent events and 1,305 temporal encounters with 237 urgent events. Continuous M2 temporal performance remained essentially unchanged: AUC 0.846 (95% CI 0.817–0.874),

Brier score 0.103, calibration intercept −0.165 (95% CI −0.255 to −0.075), and calibration slope 0.952 (95% CI 0.855–1.049).

Supplementary Table S3. Decomposition of simplification loss

Representation	Temporal AUC	Categorisation loss	Rounding loss
Continuous M2 probability model	0.845	0.000	0.000
Categorised M2, continuous coefficients	0.788	0.057	0.000
Categorised integer score	0.785	0.057	0.003

Supplementary Table S4. Counts underlying temporal specificity and negative likelihood ratios

Strategy	Threshold	Low-risk n	False negatives	True negatives	Non-urgent N	Specificity	LR−
Locked integer score	Score ≤ −1	42	1	41	1,181	3.5%	0.11
Adjacent integer score	Score ≤ 0	161	8	153	1,181	13.0%	0.24
Continuous M2 probability	p ≤ 0.0648	589	17	572	1,181	48.4%	0.14

The temporal cohort contained 1,181 non-urgent encounters (1,440 total minus 259 urgent). Specificity is true negatives divided by 1,181; LR− is (1 − sensitivity)/specificity using the corresponding unrounded proportions.

Supplementary Table S5. Sensitivity analysis removing the sparse interaction term

Model variation	Temporal AUC (95% CI)	Cal. intercept	Cal. slope	Sensitivity	Low-risk yield	Complete-card reproducibility	Unique cards / 800
Standard M2 with interaction	0.845 (0.818–0.872)	−0.178	0.942	99.6%	2.9% (42/1,440)	0.125% (1/800); 95% CI 0.003–0.694	798
M2 without interaction	0.844 (0.816–0.871)	−0.171	0.948	99.6%	2.8% (40/1,440)	26.5% (212/800); 95% CI 23.5–29.7	102

Supplementary Table S6. Final development score card and calibration parameters

Predictor	Clinical definition	Coefficient β	β/B (B = 0.40)	Rounded points
Age	≥50 years	0.381102	0.953	+1
CRP	≥50 mg/L	1.250314	3.126	+3
WBC	≥12 × 10^9/L	1.102111	2.755	+3
Temperature	≥38 °C	1.344205	3.361	+3
Shock index	≥0.9	1.228103	3.070	+3
Absent ovarian flow	Present	0.781204	1.953	+2
Adnexal mass	Present	0.445102	1.113	+1
Complex free fluid	Present	0.249811	0.625	+1
Peritonism	Present	1.277301	3.193	+3
Positive beta-hCG	Present	0.411203	1.028	+1
beta-hCG × complex fluid	Interaction present	0.264102	0.660	+1
Tubal-wall thickening	Present	−0.312104	−0.780	−1
Haematuria	Present	0.045101	0.113	0
Hydronephrosis on US	Present	−0.055102	−0.138	0

Points = sign(β) × floor(|β|/0.40 + 0.5). Attainable primary score range −1 to +22. Locked development rule-out threshold: score ≤ −1. Probability mapping: logit(p) = −3.714201 + 0.448112 × score. B = 0.40 was the prespecified primary scaling constant; reviewer-requested post hoc sensitivity analyses at B = 0.30 and B = 0.50 are reported in Tables S16–S17.

The intercept of the categorical M2 coefficient model is −3.811204; it is not assigned integer points. Full-precision coefficients determine point assignments, including complex free fluid β = 0.249811 (one point at B = 0.30 and 0.40; zero points at B = 0.50).

Supplementary Table S6a. Inter-reader reproducibility and reader-specific model discrimination

Panel A. Original paired 2×2 reader classifications and agreement statistics.

Descriptor	Both +	R1 only	R2 only	Both −	Agreement	κ (95% CI)
Complex free fluid	132	51	55	722	89.0%	0.645 (0.583–0.707)
Absent ovarian flow	58	15	19	868	96.5%	0.754 (0.675–0.833)
Adnexal mass	124	45	51	740	90.0%	0.660 (0.597–0.723)
Tubal-wall thickening	78	31	35	816	93.1%	0.664 (0.588–0.739)
Hydronephrosis on US	124	26	30	780	94.2%	0.781 (0.726–0.836)

Panel B. Discrimination after reader-specific substitution into the locked M2 model.

**Ultrasound reading source**	AUC (patient-cluster bootstrap 95% CI)
Routine clinical reading	0.845 (0.798–0.892)
Reader 1	0.852 (0.804–0.900)
Reader 2	0.841 (0.791–0.891)

The reader substudy used an outcome-stratified random sample of 960 ultrasound examinations drawn exclusively from the temporal-evaluation period (192 urgent; 768 non-urgent), corresponding to temporal-period sampling fractions of 74.1% and 65.0%, respectively. The 960 examinations belonged to 874 unique patients (795 with one examination, 72 with two, and 7 with three; maximum 3). All five 2×2 tables contain the same n = 960 paired readings. Cohen's kappa confidence intervals are asymptotic intervals calculated from the supplied paired counts and are interpreted as sample-specific because no prevalence reweighting was applied. Reader-specific AUCs use the locked development M2 coefficients without refitting. The primary uncertainty estimates shown in Panel B are patient-cluster bootstrap 95% CIs accounting for repeated examinations within 79 patients. For comparison, the original examination-level DeLong 95% CIs were 0.801–0.889 for routine clinical reading, 0.808–0.896 for Reader 1 and 0.796–0.886 for Reader 2; the close agreement shows that clustering modestly widened uncertainty without changing the substantive conclusion.

Supplementary Table S7. Locked continuous model specifications

Predictor	Unit / coding	M1	M2	M3
Intercept	Constant	−4.502154	−4.811205	−5.106214
Age	(Age [years] − 39.0) / 15.2	0.041022	0.044102	0.042011
CRP	(CRP [mg/L] − 7.2) / 45.4	0.012105	0.011032	0.009022
WBC	(WBC [10^9/L] − 8.6) / 3.4	0.105114	0.092105	0.088045
Temperature	(Temperature [°C] − 36.9) / 0.6	0.410218	0.402114	0.381105
Shock index	(Shock index − 0.6) / 0.12	0.180112	0.175022	0.166114
Positive beta-hCG	1 = yes	1.250322	0.612045	0.580122
Haematuria	1 = yes	0.115045	0.080011	0.041011
Peritonism	1 = yes	1.455211	1.302115	1.185202
Complex free fluid	1 = yes	—	0.510202	0.450114
Absent ovarian flow	1 = yes	—	0.880154	0.812205
Adnexal mass	1 = yes	—	0.465112	0.405022
Tubal-wall thickening	1 = yes	—	−0.355122	−0.310115
Hydronephrosis on US	1 = yes	—	−0.075045	−0.060102
beta-hCG × free fluid	1 = yes	—	0.315022	0.288045
Perinephric stranding	1 = yes	—	—	0.820114
Bowel-wall discontinuity	1 = yes	—	—	1.450221
Mesenteric fat stranding	1 = yes	—	—	0.610115
Free extraluminal gas/fluid	1 = yes	—	—	1.105202
Haematuria × stranding	1 = yes	—	—	0.415112

Predicted probability is $p = 1/[1 + \exp(-LP)]$, where LP is the intercept plus the sum of the coefficients multiplied by the explicitly coded predictors in this table. Continuous terms use the centred and scaled variables shown, rather than untransformed measurements. Binary predictors use 0 = absent and 1 = present; interaction terms are products of their binary components. For the reference profile Age = 39, CRP = 7.2, WBC = 8.6, Temperature = 36.9 and Shock index = 0.6, with all binary terms zero, the model probabilities are 0.01096356 (M1), 0.00807235 (M2) and 0.00602249 (M3).

Supplementary Table S8. Mutually exclusive clinical diagnoses and outcome mapping

Diagnosis category	Urgent status	Overall n
Adnexal torsion	Urgent	120
Appendicitis requiring urgent management	Urgent	375
Bowel obstruction, perforation or ischaemia	Urgent	195
Ectopic pregnancy requiring intervention	Urgent	48
Infected obstructed urinary tract	Urgent	139
Vascular emergency	Urgent	54
of which ovarian vein thrombosis	(nested)	39
of which pelvic thrombophlebitis / deep pelvic venous thrombosis	(nested)	9
of which spontaneous vessel rupture or bleeding AVM	(nested)	6
Severe PID requiring surgical drainage	Urgent	0
Haemodynamically significant haemorrhagic ovarian cyst	Urgent	0
**Total urgent pathology**	**Urgent**	**931**
Benign or nonspecific pain	Non-urgent	1,546
Other non-urgent gastrointestinal or genitourinary	Non-urgent	1,148
PID without abscess	Non-urgent	464
Uncomplicated renal colic or urinary tract infection	Non-urgent	711
**Total non-urgent pathology**	**Non-urgent**	**3,869**
**Total cohort**	**All**	**4,800**

The three indented rows are nested within the 54 vascular emergencies and must not be added to the column total. The two zero-count rows represent narrowly defined intervention-linked categories and should not be read as absence of PID or ovarian-cyst presentations from the source population.

Supplementary Table S9. Post-hoc sample-size context

Model	Criterion	Inputs	Minimum N	Observed N	Ratio
M2	Target shrinkage ≥ 0.90	P = 14, R^2cs = 0.276	**383**	3,360	**8.77**
M2	R^2app − R^2adj ≤ 0.05	P = 14, R^2cs = 0.276	371	3,360	9.06
M2	Risk margin of error 0.05	prevalence 0.20	246	3,360	13.66
M2	Events per parameter	672 events / 14	—	—	48.0
M3	Events per parameter	265 events / 19	—	—	13.9

The binding requirement is the target-shrinkage criterion at 383. Maximum attainable Cox–Snell R^2 at a prevalence of 0.20 is 0.632, so the apparent Cox–Snell R^2 of 0.276 observed in the development data corresponds to a Nagelkerke R^2 of 0.436. The value 0.276 was used solely for this illustrative post-hoc sample-size context analysis and was not an anticipated design input. These calculations address global model adequacy and were not interpreted as criteria for the stability of individual rounded point weights.

Supplementary Table S10. Encounters and urgent events by calendar year

Year	Encounters	Urgent events	Prevalence	Period	Cumulative
2013	450	82	18.2%	Development	
2014	460	101	22.0%	Development	
2015	480	91	19.0%	Development	
2016	490	103	21.0%	Development	
2017	500	94	18.8%	Development	
2018	510	101	19.8%	Development	
2019	470	100	21.3%	Development	3,360 / 672
2020	390	65	16.7%	Temporal	
2021	520	94	18.1%	Temporal	
2022	530	100	18.9%	Temporal	1,440 / 259

Development years sum to 3,360 encounters and 672 urgent events; temporal years to 1,440 and 259, after removal of the 212 overlapping encounters. Annual prevalence ranged from 18.2% to 22.0% during development (SD 1.43 percentage points) and fell to 16.7% in 2020 before partial recovery. This table documents that the development–temporal split follows calendar period alone.

Supplementary Table S11. Penalty selection and single-penalty sensitivity analysis

Item	M1	M2	M3	Selection rule
Selected lambda	0.019	0.023	0.035	Minimum cross-validated deviance
Inter-imputation range	—	0.018–0.028	—	10-fold, patient clusters intact
Binary predictors	Standardised	Standardised	Standardised	—
Intercept	Unpenalised	Unpenalised	Unpenalised	—
Single-lambda sensitivity: temporal AUC	—	0.844 (0.817–0.871)	—	lambda selected once in stacked imputed data
Single-lambda sensitivity: card reproducibility	—	0.375% (3/800); 95% CI 0.08–1.09	—	800 resamples

Rubin's rules have no exact analogue for parameter pooling after separately tuned penalised models. The single-lambda sensitivity analysis therefore evaluated whether per-imputation penalty selection materially affected the results. Fixing the penalty at a single value left temporal discrimination essentially unchanged (0.844 versus 0.845) and increased exact card reproducibility only from 0.125% to 0.375%, whereas removing the sparse interaction increased reproducibility to 26.5%.

Standard M2 with per-imputation tuning reproduced the original card in 1/800 resamples (0.125%; 95% CI 0.00–0.69), compared with 3/800 (0.375%; 0.08–1.09) using a fixed lambda and 212/800 (26.5%; 23.5–29.7) after removal of the sparse interaction. All three variants retained a temporal AUC of 0.844–0.845. These results suggest that variability in penalty selection was not the principal explanation for score-card instability; instability was substantially reduced only after removal of the sparse interaction term.

Supplementary Table S12. Ungrouped temporal operating profile of the integer score

Temporal cohort, every observed integer score (main Table 4 groups scores above 6). For descriptive tabulation, each incomplete categorical predictor is replaced by its modal category across the 20 imputations for that encounter, followed by application of the single fixed card.

Score	n	Events	Observed risk	Mapped risk	Cum. n	Cum. FN	Sens.	Spec.	NPV
-1	42	1	2.38%	1.53%	42	1	99.6%	3.5%	97.6%
0	119	7	5.88%	2.38%	161	8	96.9%	13.0%	95.0%
1	251	15	5.98%	3.68%	412	23	91.1%	32.9%	94.4%
2	217	17	7.83%	5.64%	629	40	84.6%	49.9%	93.6%
3	178	19	10.67%	8.55%	807	59	77.2%	63.3%	92.7%
4	142	20	14.08%	12.77%	949	79	69.5%	73.7%	91.7%
5	112	20	17.86%	18.64%	1061	99	61.8%	81.5%	90.7%
6	88	20	22.73%	26.40%	1149	119	54.1%	87.2%	89.6%
7	65	18	27.69%	35.95%	1214	137	47.1%	91.2%	88.7%
8	55	18	32.73%	46.77%	1269	155	40.2%	94.3%	87.8%
9	42	18	42.86%	57.90%	1311	173	33.2%	96.4%	86.8%
10	33	18	54.55%	68.29%	1344	191	26.3%	97.6%	85.8%
11	26	15	57.69%	77.12%	1370	206	20.5%	98.6%	85.0%
12	17	10	58.82%	84.07%	1387	216	16.6%	99.2%	84.4%
13	11	7	63.64%	89.20%	1398	223	13.9%	99.5%	84.0%
14	8	6	75.00%	92.82%	1406	229	11.6%	99.7%	83.7%
15	7	6	85.71%	95.29%	1413	235	9.3%	99.7%	83.4%
16	6	5	83.33%	96.94%	1419	240	7.3%	99.8%	83.1%
17	5	4	80.00%	98.02%	1424	244	5.8%	99.9%	82.9%
18	4	4	100.00%	98.73%	1428	248	4.2%	99.9%	82.6%
19	4	4	100.00%	99.18%	1432	252	2.7%	99.9%	82.4%
20	3	3	100.00%	99.48%	1435	255	1.5%	99.9%	82.2%
21	3	2	66.67%	99.67%	1438	257	0.8%	100.0%	82.1%
22	2	2	100.00%	99.79%	1440	259	0.0%	100.0%	82.0%

Cumulative n reaches 1,440 and cumulative events 259. Specificity reaches 100% at score 21, when all 1,181 non-urgent encounters have been included. This is a descriptive modal-profile distribution and does not reproduce the across-imputation pooling used for Table 2; it does not define a new threshold. Mapped risks use $\text{logit}(p) = -3.714201 + 0.448112 \times \text{score}$.

Supplementary Table S13. Development–temporal case-mix comparison using mean ± SD and standardized mean differences

Variable	Development	Temporal	SMD
Age, y	41.2 ± 15.1	42.2 ± 15.2	−0.066
CRP, mg/L	22.4 ± 35.2	20.6 ± 36.5	+0.050
WBC, ×10⁹/L	9.10 ± 3.40	8.96 ± 3.50	+0.040
Temperature, °C	36.95 ± 0.55	36.95 ± 0.58	−0.005
Shock index	0.65 ± 0.12	0.65 ± 0.13	−0.005

SMDs compare the development and temporal cohorts on the original continuous scale. All absolute SMDs were ≤0.066, indicating only small shifts in these measured case-mix dimensions; this does not exclude unmeasured temporal drift.

Supplementary Table S14. Development distribution of the integer score

Score	N	Events	Observed event rate
−1	104	4	3.85%

Score	N	Events	Observed event rate
0	635	36	5.67%
1	415	28	6.75%
2	180	16	8.89%
3	450	53	11.78%
4	310	45	14.52%
5	140	25	17.86%
6	280	64	22.86%
7	210	60	28.57%
8	120	42	35.00%
9	150	63	42.00%
10	90	44	48.89%
11	70	40	57.14%
12	50	32	64.00%
13	40	27	67.50%
14	30	22	73.33%
15	25	19	76.00%
16	20	16	80.00%
17	15	13	86.67%
18–22	26	23	88.46%
**Total**	**3,360**	**672**	**20.00%**

Counts are source-verified and sum to 3,360 development encounters and 672 urgent events (20.0%). The non-monotonic distribution of encounter counts across adjacent integer scores reflects the combinatorial structure of the score card, including multiple +3-point components, rather than a violation of the prespecified thresholding procedure. Scores 18–22 are grouped because individual cells are sparse.

Supplementary Table S15. Empirical coefficient uncertainty and integer point-assignment stability

Component	β	Points	Bootstrap SE	Nearest B=0.40 boundary	|β−boundary|	Distance / SE	Changed point
Age ≥50 years	+0.381102	+1	0.101	+0.20	0.181102	1.79	15.1%
CRP ≥50 mg/L	+1.250314	+3	0.112	+1.40	0.149686	1.34	21.4%
Complex free fluid	+0.249811	+1	0.052104	+0.20	0.049811	0.96	40.2%
Adnexal mass	+0.445102	+1	0.121	+0.60	0.154898	1.28	16.5%
Peritonism	+1.277301	+3	0.115	+1.40	0.122699	1.07	19.4%
WBC ≥12 × 10^9/L	+1.102111	+3	0.131	+1.00	0.102111	0.78	28.6%
Absent ovarian flow	+0.781204	+2	0.170	+0.60	0.181204	1.07	30.1%
Tubal-wall thickening	−0.312104	−1	0.145	-0.20	0.112104	0.77	24.3%
Positive beta-hCG	+0.411203	+1	0.185	+0.60	0.188797	1.02	37.8%
Temperature ≥38 °C	+1.344205	+3	0.260	+1.40	0.055795	0.21	12.3%
Shock index ≥0.9	+1.228103	+3	0.315	+1.40	0.171897	0.55	18.8%
beta-hCG × complex fluid	+0.264102	+1	0.365	+0.20	0.064102	0.18	58.5%

Empirical bootstrap SEs are from the full-pipeline resamples. Distance/SE is a descriptive boundary-proximity index, not a hypothesis test. Source-verified point-assignment change frequencies from the original 800 patient-cluster bootstrap resamples are reported in the final column. The interaction changed point assignment in 468/800 (58.5%) resamples. For the two zero-point components not shown in the table, point assignment changed in 68/800 (8.5%) resamples for haematuria and 90/800 (11.2%) for hydronephrosis on ultrasound. Across 800 full-pipeline resamples, 798 unique cards were generated and the original card was reproduced in 1/800 (0.125%); without the interaction there were 102 unique cards and exact reproduction rose to 212/800 (26.5%).

Supplementary Table S16. Sensitivity of integer-score performance and stability to the scaling constant B

Panel A. Discrimination, development threshold and score-card stability.

B	Attainable score range	Development threshold	Development sensitivity	Categorical M2 AUC	Integer-score AUC (95% CI)	Rounding loss	Unique cards / 800	Exact reproduction
0.30	−1 to +28	≤0	96.6% (649/672)	0.788	0.786 (0.754–0.816)	0.002	785	4/800 (0.50%)
0.40 (primary)	−1 to +22	≤−1	99.4% (668/672)	0.788	0.785 (0.753–0.815)	0.003	798	1/800 (0.125%)
0.50	−1 to +19	≤−1	98.8% (664/672)	0.788	0.781 (0.749–0.811)	0.007	799	0/800 (0.00%)

Panel B. Temporal operating performance at the development-locked threshold.

B	Low-risk N	Yield	False negatives	Sensitivity	Specificity	NPV	LR−
0.30	108	7.5%	10	96.1%	8.4%	90.7%	0.46
0.40 (primary)	42	2.9%	1	99.6%	3.5%	97.6%	0.11
0.50	58	4.0%	4	98.5%	4.6%	93.1%	0.34

Alternative B values were evaluated post hoc in response to peer review and were not used to select the primary score. Across B = 0.30, 0.40 and 0.50, rounding-related AUC loss remained small (0.002–0.007), whereas exact complete-card reproduction remained ≤0.50%. Operational thresholds and low-risk yield varied with scaling, underscoring that the integer representation is not invariant to B.

Development low-risk counts/false negatives were 272/23 at B = 0.30, 104/4 at B = 0.40 and 141/8 at B = 0.50. Temporally, 42 encounters (one event) were low risk under both B = 0.40 and 0.50; an additional 16 encounters (three events) were low risk only at B = 0.50. Their scored profile included tubal-wall thickening and complex free fluid with all other positively scored findings absent: the score changed from 0 to −1 because complex free fluid changed from one point to zero. The reported ranges are theoretical attainable ranges, not observed maxima.

Supplementary Table S17. Point assignments and bootstrap point-change frequencies across scaling constants

Component	β	B=0.30 points	Changed %	B=0.40 points	Changed %	B=0.50 points	Changed %
Age ≥50 years	0.381102	+1	18.4%	+1	15.1%	+1	42.5%
CRP ≥50 mg/L	1.250314	+4	29.2%	+3	21.4%	+3	51.2%
WBC ≥12 × 10^9/L	1.102111	+4	15.5%	+3	28.6%	+2	36.4%
Temperature ≥38 °C	1.344205	+4	22.1%	+3	12.3%	+3	27.5%
Shock index ≥0.9	1.228103	+4	24.8%	+3	18.8%	+2	44.6%
Absent ovarian flow	0.781204	+3	35.6%	+2	30.1%	+2	48.1%
Adnexal mass	0.445102	+1	45.3%	+1	16.5%	+1	25.3%
Complex free fluid	0.249811	+1	31.1%	+1	40.2%	0	34.4%
Peritonism	1.277301	+4	18.6%	+3	19.4%	+3	45.8%
Positive β-hCG	0.411203	+1	36.2%	+1	37.8%	+1	40.5%
β-hCG × complex free fluid	0.264102	+1	32.4%	+1	58.5%	+1	76.5%
Tubal-wall thickening	−0.312104	−1	10.5%	−1	24.3%	−1	31.7%
Haematuria	0.045101	0	12.2%	0	8.5%	0	18.1%
Hydronephrosis on US	−0.055102	0	21.4%	0	11.2%	0	24.2%

For the β-hCG × complex-free-fluid interaction (β = 0.264), the distance to the nearest rounding boundary was 0.114 at B = 0.30 (boundary 0.15), 0.064 at B = 0.40 (boundary 0.20), and 0.014 at B = 0.50 (boundary 0.25). Corresponding point-change frequencies were 32.4%, 58.5% and 76.5%, respectively. These results demonstrate increasing boundary sensitivity as B approaches the interaction coefficient.

Supplementary Note S1. Interpretation boundaries

Primary performance estimates are conditional on the observed retrospective cohort, the stated predictor definitions, the penalised modelling pipeline, the prespecified B = 0.40 scaling constant and the locked thresholds. Reviewer-requested post hoc sensitivity analyses at B = 0.30 and B = 0.50 showed that the small rounding-related discrimination loss and poor complete-card reproducibility persisted across reasonable changes in scaling, but operational thresholds, yield and individual point assignments remained B-dependent. These sensitivity analyses test robustness and do not identify an optimal scaling constant.

The score ≤ −1 threshold corresponds to the minimum attainable score and can occur only when tubal-wall thickening is present and all eleven positively weighted components are absent; the two zero-weighted components do not affect this classification. An encounter with none of the scored findings present scores 0 and is therefore not classified as low risk.

The descriptive modal-profile score distribution shows lower-score observed-versus-mapped risk discrepancies, including 7/119 events at score 0 (5.88% versus 2.38%). It is distinct from the global

calibration statistics pooled across imputations (intercept −0.109, slope 1.013) and from the mean-probability spline in main Figure 4. These three summaries should not be treated as interchangeable estimates from a single score distribution.

This study does not establish that either the continuous model or the integer score can be used to autonomously defer ultrasound, CT or MRI, hospital admission, specialist assessment or therapeutic intervention. Independent multicentre external evaluation and prospective workflow assessment are required before any clinical implementation.

Supplementary Figures

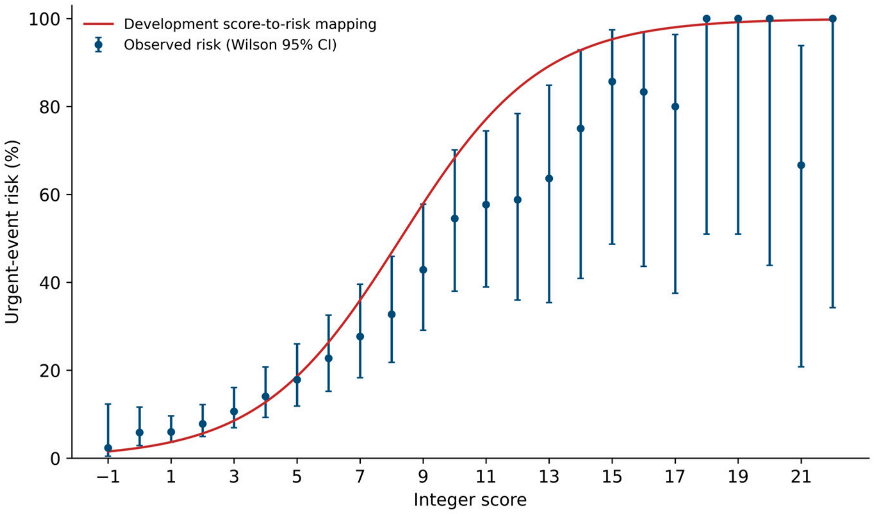

Supplementary Figure S1. Mapped versus observed risk by integer score in the descriptive modal-profile temporal distribution (Table S12). Points show observed event rates with 95% Wilson confidence intervals; the fitted development score-to-risk mapping is shown as a continuous curve. The largest clinically relevant discrepancy at the rule-out end occurs at score 0 (7/119 events; 5.88% observed versus 2.38% mapped).

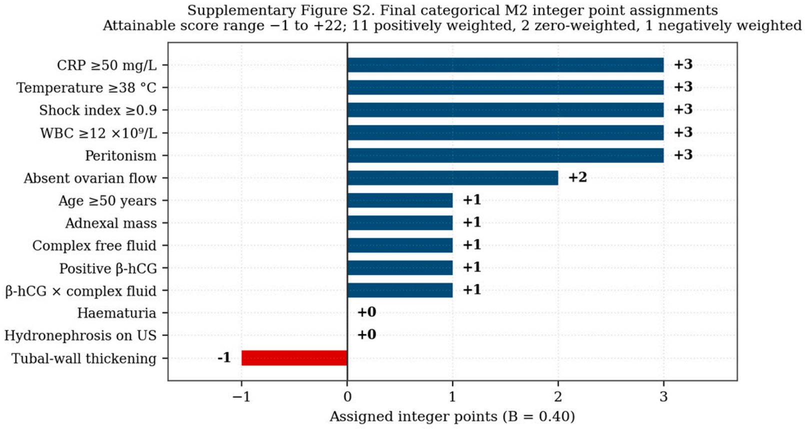

Supplementary Figure S2. Final categorical M2 integer point assignments under B = 0.40. Eleven components carry positive weight, two carry zero weight and one carries −1.
